# The species chromatogram, a new graphical method to represent, characterize, and compare the ecological niches of different species

**DOI:** 10.1002/ece3.8830

**Published:** 2022-04-13

**Authors:** Loïck Kléparski, Grégory Beaugrand

**Affiliations:** ^1^ UMR 8187 ‐ LOG ‐ Laboratoire d’Océanologie et de Géosciences Université du Littoral Côte d'Opale CNRS Université de Lille Wimereux France; ^2^ Marine Biological Association Plymouth UK

**Keywords:** degree of niche overlapping, ecological niche, gradient analysis, hypervolume, niche breadth, niche optimum

## Abstract

The ecological niche *sensu* Hutchinson is defined as the set of environmental conditions allowing a species to grow, maintain, and reproduce. This conception of the niche, which is assimilated to a *p*‐dimensional hypervolume, with *p* representing all environmental variables, has been widely applied in ecology. However, displaying the niche hypervolume has proved challenging when more than three environmental dimensions are considered simultaneously. We propose a simple method (implemented in the *specieschrom* R package) that displays the full multidimensionality of the ecological niche of a species into a two‐dimensional space by means of a graphic we call species chromatogram. This method gives a graphical summary of the niche by representing together abundance gradients with respect to all environmental variables. A chromatogram enables niche optimums and breaths to be rapidly quantified, and when several chromatograms are examined (one per species), rapid comparisons can be made. From our chromatograms, we proposed a procedure that quantifies niche optimum and breadth as well as niche overlapping (index *D*) and the identification of the most discriminant combination of environmental variables. We apply these analyses on eight planktonic species collected by the Continuous Plankton Recorder (CPR) survey in the North Atlantic Ocean using 10 environmental variables. We display their full multidimensional niches and quantify their niche optimums and breadths along each dimension. We also compare our index *D* with other indices by means of *hypervolume* and *dynRB* R packages. By catching the full complexity of the niche, species chromatograms allow many different niche properties to be rapidly assessed and compared among species from niche optimums and breadths to the identification of the most relevant environmental parameters and the degree of niche overlapping among species. Species chromatograms may be seen as species’ fingerprint and may also allow a better identification of the mechanisms involved in species assembly.

## Graphical abstract

In this article, we present a method (called species chromatogram) that displays and quantifies the (multidimensional) ecological niche of a species. In addition, the method quantifies niche optimums and breadths along each niche dimensions and estimates a degree of niche overlapping among species.

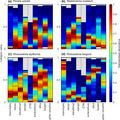

## 1 INTRODUCTION

Throughout the 20th century, various definitions of the concept of ecological niche have been proposed. The first was formulated in 1917 by Joseph Grinnell, who defined the niche as the place occupied by a species in an environment (Grinnell, 1917). Ten years later, in 1927, Charles Elton proposed a more functional concept, the niche being seen as the role of a species in the food chain and its influence on the environment (Elton, 1927). These two conceptions envisioned the niche as an attribute of the environment and not as a property of a species, the niche being the place or the role that a species plays within a community and/or an ecosystem (Colwell & Rangel, 2009; Pulliam, 2000).

In 1957, Evelyn Hutchinson proposed a new concept of the niche, envisioned here as a species property (Hutchinson, 1957). He defined the niche as a set of environmental variables enabling a species to grow, maintain, and reproduce. According to Hutchinson, the niche of a species can be viewed as a *p*‐dimensional hypervolume in which each environmental combination enables a species to exist indefinitely (i.e., the species fundamental niche), this hypervolume being subsequently modulated by species interactions (i.e., the realized niche; Hutchinson, 1978). This way to define the niche was in line with the law of tolerance, which states that a species is limited by its range of tolerance for environmental factors (Shelford, 1913).

A corollary of this new concept is that two species with the same niche in the same location cannot coexist, a statement known as the principle of competitive exclusion (Gause, 1934; Hutchinson, 1978). Therefore, each species of a community has a unique niche and the niche–environment interaction determines the place where a species lives and when it is active (Beaugrand, 2015). Hence, the niche is a powerful tool to explain major biogeographical patterns at the species and even at the community levels (Beaugrand et al., 2020) because of the reciprocal correspondence, called Hutchinson's duality, between the niche space and the real physical space (Colwell & Rangel, 2009). Hutchinson's niche concept has been used to assess species and community responses to climate change in both space and time (Araújo & Guisan, 2006; Goberville et al., 2015; Thuiller et al., 2009).

However, the clear representation of the multidimensionality of the niche is challenging because of the difficulty for human to handle a space beyond more than three dimensions. Mathematicians have developed tools to solve this problem, e.g., Schlegel's diagrams, which enable the projection of a four‐dimensional hypercube (i.e., a tesseract) into a three‐dimensional space, in other words the representation of a *p*‐dimensional polytope into a *p*‐1‐dimensional space. In ecology, indirect and direct gradient analyses have been applied but these techniques have some limitations due to normality assumption, the lack of explanatory power of the components, or inherent complexity (Beaugrand et al., 2000; Ter Braak & Prentice, 1988). Most of the time, ecologists manage dimensionality by seeking to summarize the information in a limited set of dimensions. To do so, they use multivariate analyses (e.g., principal component analysis (PCA)) to characterize and display the niche (e.g., Broennimann et al., 2012). The Outlying Mean Index (OMI) is another technique that is also applied to characterize some properties of the niche (e.g., niche breadth) and assess which environmental factors are the most structuring in a community (Dolédec et al., 2000). However, interpreting the outputs of those techniques is often challenging because the resulting components that are used to display the niche are typically a linear combination of different environmental dimensions and some variables can contribute to more than one principal component.

**FIGURE 1:**
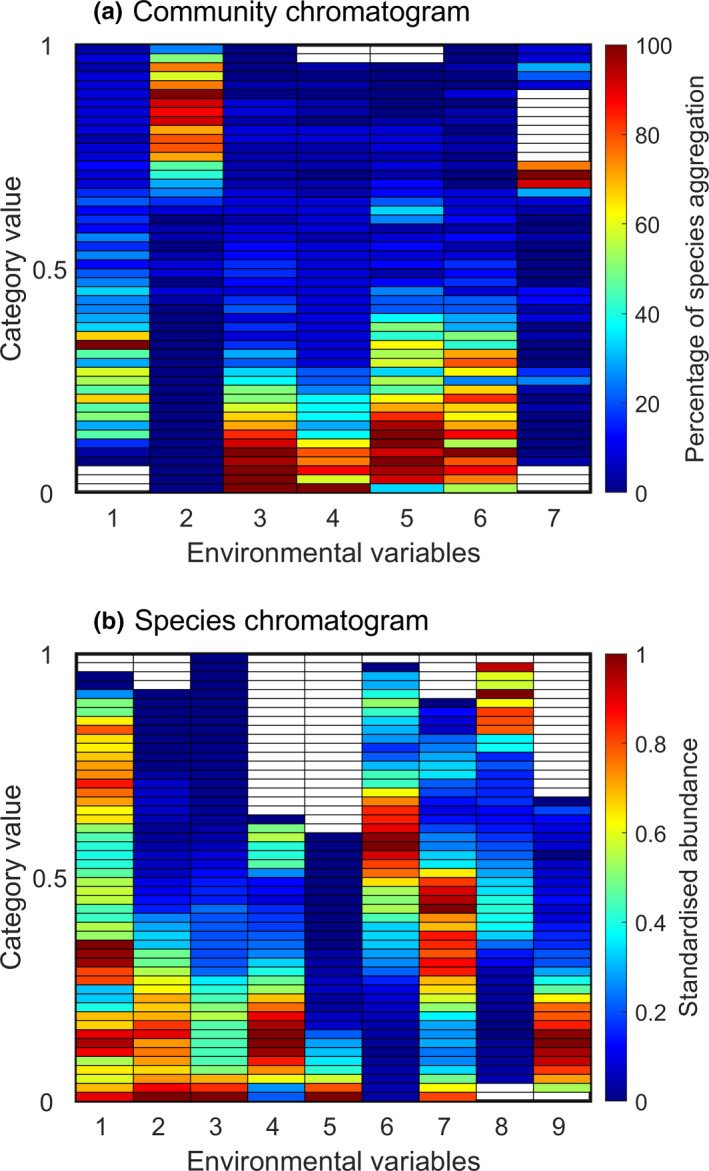
Chromatogram of a hypothetical community (a) and a virtual species (b). (a) A community chromatogram shows where species of an assemblage aggregate along multiple ecological dimensions. Each column represents an environmental gradient divided into α categories (see Materials and Methods), from the lowest values taken by an environmental variable (bottom categories) to the highest (top categories). The color in a category denotes the percentage of species of an assemblage, between 0 and 100%. Blue color indicates that no or few species of an assemblage are found in a category and red color indicates that the majority of the species composing an assemblage are found in a category. In this hypothetical example, large bands of high aggregation (see Table S1 for a definition) are observed from dimensions 3 to 5 and narrow bands for dimension 7. (b) A species chromatogram displays the multidimensional niche of a species into a two‐dimensional space. Each column represents an environmental gradient divided into α categories, from the lowest (bottom categories) to the highest values taken by an environmental variable (top categories). The color in a category denotes the standardized abundance of a species between 0 and 1. Blue color in a category means that the species has a nil or low abundance in a category and red color means that the species has a high abundance in a category. In this hypothetical example, large colored bands of high abundance are observed for environmental dimension 1 and narrower bands for environmental dimensions 5 and 9

The niche hypervolume can also be represented by a set of two‐dimensional pair plots of all possible combinations of the *p*‐environmental variables defining the niche hyperspace. However, this method leads to a vast amount of figures for a single species when the number of environmental dimensions is large (Blonder et al., 2014). Recently, Kléparski et al. (2021) proposed a new method called “the environmental chromatogram” to represent graphically the environmental signature of plankton assemblages in the North Atlantic Ocean, with color bands representing the percentage of species aggregation within an assemblage or a community along multiple environmental gradients (Kléparski et al., 2021; Figure 1a). The method allowed the authors to rapidly display the optimal environmental conditions in which an assemblage was found. Applied at a community/assemblage level, we propose to call such a graphic a community chromatogram from now on (see Table S1 for a full definition of the terms used in this paper).

In this study, we adapt this method at a species level to characterize graphically the ecological niche of a species by projecting the multidimensional space into a plane. Here, the resulting graphic is termed a species chromatogram (Figure 1b and Table S1). From species chromatograms, we propose a way to measure (i) niche optimum and (ii) breadth, (iii) to quantify the degree of niche overlapping among species, and (iv) to identify the most discriminant combinations of environmental variables in term of niche differentiation. We apply the procedures on real species, i.e., four phytoplankton and four zooplankton species/taxa routinely sampled by the Continuous Plankton Recorder (CPR) survey in the North Atlantic Ocean. Finally, using 14 pseudo‐species, we compared our estimation of niche overlapping against values obtained by means of the *hypervolume* (Blonder et al., 2014, 2018) and *dynRB* (Junker et al., 2016) R packages.

## 2 MATERIALS AND METHODS

### 2.1 Materials

Plankton abundance data came from the Continuous Plankton Recorder (CPR) survey (Batten et al., 2003). It is a long‐term plankton monitoring program currently operated by the Marine Biological Association of the United Kingdom. Started in 1931, the program has sampled plankton on a monthly basis in the North Atlantic Ocean and its adjacent seas. The CPR machine is a high‐speed plankton recorder towed behind voluntary merchant ship, called “ship of opportunity,” and operating at a depth of approximately ~7–10 m (Hays & Warner, 1993; Warner & Hays, 1994). We chose four diatoms and four copepods to test whether the robustness of our methods did not vary with taxonomic group. For each taxon, we chose species with known different spatial distribution (Barnard et al., 2004). Selected diatoms were *Paralia sulcata* (neritic tychopelagic species), *Skeletonema costatum* (neritic), *Rhizosolenia styliformis* (eurygraph), and *R*. *bergonii* (oceanic). Chosen copepods were *Temora longicornis* (temperate neritic species), *Clausocalanus* spp. (warm temperate oceanic), *Calanus finmarchicus* (subarctic oceanic), and *Calanus helgolandicus* (pseudo‐oceanic temperate). We used data collected between 1998 and 2018 in the North Atlantic Ocean and its adjacent seas (Helaouët, 2021). This time period was preferred to correspond to the period covered by the environmental datasets described below.

Mass concentration of chlorophyll‐a in sea water (mg m^−3^), nitrate, phosphate, and silicate concentration (mmol m^−3^) data originated from the Global Ocean Biogeochemistry Hindcast (GLOBAL_REANALYSIS_BIO_001_029) and were provided by the Copernicus Marine Environment Monitoring Service (CMEMS) (http://marine.copernicus.eu). Daily means were provided on a 0.25° resolution grid and along 75 depth levels from 0 to 5500m. The dataset covers the time period from 1993 to present and is regularly updated.

Sea water potential temperature (°C), salinity (no unit), and Mixed Layer Depth (MLD, m) data originated from the Global Ocean Ensemble Physics Reanalysis (GLOBAL_REANALYSIS_PHY_001_031) and were provided by the Copernicus Marine Environment Monitoring Service (CMEMS) (http://marine.copernicus.eu). Daily means were provided on a 0.25° resolution grid along 75 depth levels from 0 to 5500 m. The dataset covers the time period from 1993 to present and is regularly updated.

Euphotic depth data (m) originated from the Global ocean low and mid trophic levels biomass content hindcast (GLOBAL_MULTIYEAR_BGC_001_033) provided by the Copernicus Marine Environment Monitoring Service (CMEMS) (http://marine.copernicus.eu). Daily means were provided on a 0.083° resolution grid, covering the time period 1998–2020.

Photosynthetically Active Radiations clear sky in surface (PAR, in J m^−2^) originated from the ERA interim dataset provided by the European Centre for Medium‐Range Weather Forecasts (ECMWF; https://www.ecmwf.int/). Hourly means were provided on a 0.25° resolution grid, covering the time period 1998–2018. Daily PAR was estimated by summing all the values corresponding to a given day and were subsequently converted into E m^−2^ day^−1^.

*K_d_* (PAR) data originated from the Glob Colour project (https://hermes.acri.fr/). The product merges together all the daily data from satellites (MODIS, SeaWIFS, and VIIRS) available for each parameter, from September 1997 to present, and on a 4 km resolution spatial grid. It provides daily means for each parameter. As the data can be very holey because of cloud cover and sun glint effect during the winter season, missing *K_d_* (PAR) values were first spatiotemporally interpolated and the remaining missing data (i.e., the one above 45°N in winter) were interpolated with chlorophyll‐a data according to the relationships presented in Morel et al. (2007).

PAR in depth was finally estimated from the Beer–Lambert law (Swinehart, 1962):

(1) $$I_{Z}=I_{0}e^{‐K_{d}\times Z}$$

with $I_{0}$ the PAR in surface and Z the depth (from 0 to 100 m).

Bathymetry (m) came from GEBCO Bathymetric Compilation Group 2019 (The GEBCO_2019 Grid – a continuous terrain model of the global oceans and land). Data are provided by the British Oceanographic Data Centre, National Oceanography Centre, NERC, UK, doi:10/c33m. (https://www.bodc.ac.uk/data/published_data_library/catalogue/10.5285/836f016a‐33be‐6ddc‐e053‐6c86abc0788e/). To work on the same spatial grid, *K_d_* (PAR), euphotic depth, and bathymetry were interpolated on a 0.25° latitude × 0.25° longitude grid.

We used data collected between 1998 and 2018 (i.e., from January 1, 1998, to December 31, 2018) in order to work on a common time period with respect to all biological and environmental datasets. All data were subsequently arranged on a grid covering the North Atlantic Ocean (100°W–10°E and 35°N–65°N). The dimension of all matrices (a total of 10 matrices, each matrix corresponding to an environmental variable) was 121 latitudes × 441 longitudes × 7670 days. By means of nearest‐neighbor interpolation (Wackernagel, 1995), we attributed to each CPR sample a value for each of the 10 chosen environmental variables at a depth of 8 m (except for bathymetry), a value included in the range of sampling depth of the CPR instrument (Batten et al., 2003; Hays & Warner, 1993). CPR samples with at least one missing value along a single environmental dimension were discarded from the analysis.

### 2.2 Sketch of the method

The method described below has been implemented in a R package (*specieschrom*, available on Github: https://github.com/loick‐klpr/specieschrom.git). It is also available as Matlab functions (https://github.com/loick‐klpr/Species‐chromatogram‐with‐Matlab.git).

#### 2.2.1 Assessment of the species chromatogram

**FIGURE 2:**
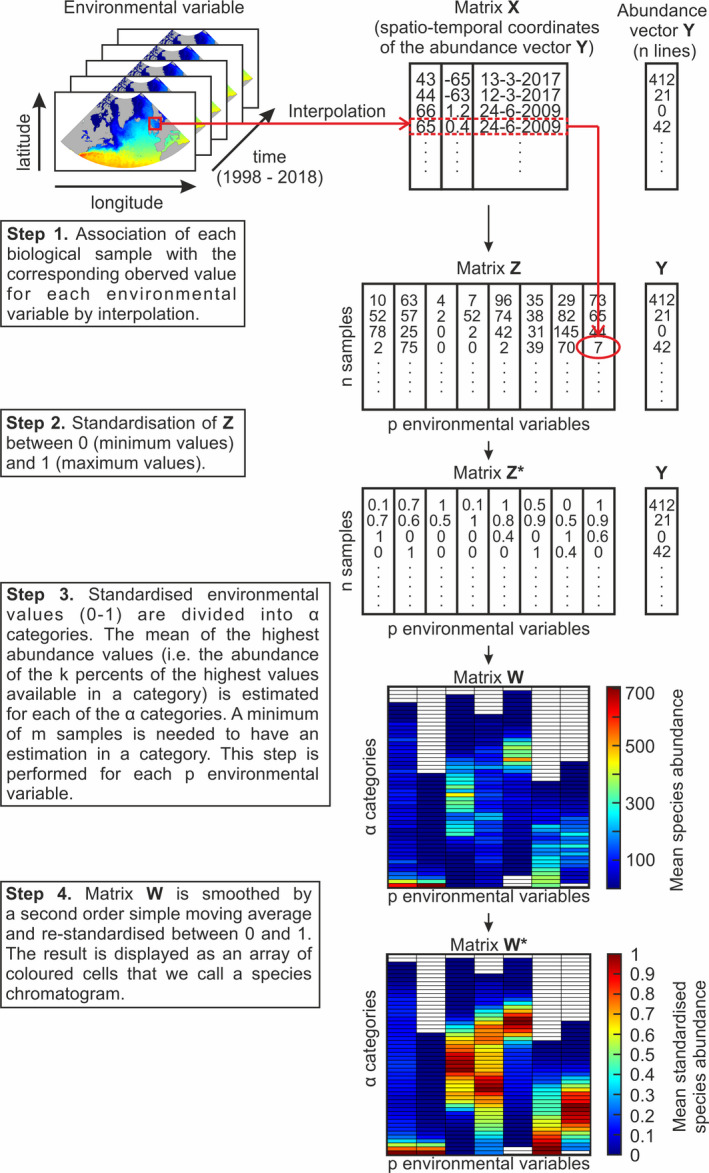
Sketch diagram summarizing the different steps leading to the representation of the multidimensional ecological niche of a species as a chromatogram

The complete procedure to build a species chromatogram was composed of four main steps (see Figure 2):

**Step 1**: Matrix **X** (with the spatiotemporal coordinates of *n* samples) and the corresponding vector **Y** (with the abundance of a species in the *n* samples) are built. Then, from *p* gridded environmental datasets, the values of the environmental variables (e.g., temperature) are assessed by nearest‐neighbor interpolation at the spatiotemporal coordinates stored in Matrix **X** (Wackernagel, 1995). This step enables the arrangement of a new matrix, Matrix **Z** (*n* samples by *p* environmental variables).

**Step 2**: Matrix **Z** is standardized between 0 (the lowest value of an environmental variable) and 1 (the highest) as follows:

(2) $$Z_{\left(i,j\right)}^{*}=\frac{Z_{i,j}‐\text{min}\left(Z_{j}\right)}{\text{max}\left(Z_{j}\right)‐\text{min}\left(Z_{j}\right)}$$

where $Z_{\left(i,j\right)}^{*}$ is the matrix of standardized environmental values for sample i and environmental variable j. Standardization is applied simultaneously along each environmental variable and for all species so that niche dimensions could be compared from one chromatogram to another.

**Step 3**: *p*‐Standardized environmental gradients are defined between 0 and 1 and divided into α equidistant categories, leading to Matrix **W**_α,p_ (α categories by *p* environmental variables). Each sample in Matrix Z* is assigned to one of the α categories along a corresponding environmental gradient in **W**. If more than *m* samples are available within a category, an estimation of maximum abundance is calculated. This calculation is done by assessing the mean of the highest abundance values only, i.e., the abundance of the *k* percents of the highest abundance values available in that category. This threshold is implemented to account for the high number of nil or low abundance in a category due to adverse environmental conditions in other dimensions; in other words, environmental conditions can be suitable in a given dimension but unsuitable in others. Such a choice is in agreement with ecological niche theory (Brown, 1984). At the end of the procedure, each column in **W** corresponds to the average of the highest abundance of a species observed along an environmental dimension (e.g., temperature, PAR) from the lowest (bottom categories in the chromatograms) to the highest (top categories) environmental values. The niche of the species is therefore displayed by the location of its abundance in the categories along each environmental gradient.

**Step 4**: For each column in **W** (i.e., each environmental gradient), a second‐order simple moving average is applied to reduce the noise in the mean abundance sometimes observed in the chromatograms from one category to another (see Figures S1–S2 vs. S3–S4 for an example with CPR data). Then, mean abundances are standardized between 0 (nil abundance) and 1 (highest abundance) for each environmental variable. Here, standardization is performed as follows:

(3) $$W_{\left(i,j\right)}^{*}=\frac{W_{\left(i,j\right)}}{\text{max}\left(W_{j}\right)}$$

where $W_{\left(i,j\right)}^{*}$ is the standardized value of Matrix **W** for sample i and environmental variable j (see Figures S3–S4 vs. S5–S6 for an example with the CPR data).

Matrix W* is finally displayed as an array of colored cells, which we call a species chromatogram, i.e., a graphic that shows how species abundance is distributed along multiple environmental gradients. (Species chromatogram can be built by means of the *chromato_env16* function available in the *specieschrom* R package.) All terms used to characterize the chromatograms are defined in Table S1.

#### 2.2.2 Niche optimum and breadth

Niche optimum along each environmental dimension is assessed for each species and each dimension (i.e., each chromatogram; Tables 1 and 2). To do so, we assume that highest species abundances are observed when environmental conditions are optimal (Brown, 1984; Helaouët & Beaugrand, 2009). For each niche dimension (i.e., each of the *p* environmental gradients/variables), the categories of the chromatogram where species abundance is maximal are identified. To provide a more precise estimation, the optimum is assessed by averaging the values of the environmental variable (Matrix **Z**) associated with the samples used to assess the species abundance within the selected category (i.e., the *k* % of the samples with the highest values available in a category).

Niche breadth (Table S1) *E* is assessed for each niche dimension *p* by calculating the percentage of categories with an abundance higher than or equal to threshold *T*, with 0 ≤ *T* ≤ 1:

(4) $$E=\frac{U_{\text{max}}‐U_{\text{min}}}{U^{*}}\times 100$$

with $U_{\text{max}}$ the highest category with an abundance ≥ *T* and $U_{\text{min}}$ the lowest category with an abundance ≥ *T* for a given environmental dimension *p*; the difference in the numerator represents the number of contiguous categories with abundance ≥ *T*. $U^{*}$ is the number of categories with an estimation (i.e., non‐missing values). Only the categories with an abundance > *T* are considered when *T* = 0. For each given environmental dimension, we fill categories between the smallest and the highest, assuming a unimodal (continuous) niche in agreement with ecological niche theory (Brown, 1984; Hutchinson, 1957).

Average niche breadth ($E_{T}$, Table S1) is estimated as follows:

(5) $$E_{T}=\frac{\sum_{i=1}^{p}E_{i}}{p}$$

where *p* is the number of environmental dimensions. (Niche optimums and breadths can be assessed by means of the *opti_eury_niche2* function available in the R package *specieschrom*.)

#### 2.2.3 Degree of niche overlapping

The degree of niche overlapping (Table S1) between two species of the same taxonomic group is assessed by means of index D, which is estimated by calculating the ratio of the part of the hypervolume of the niche common to the two species $V_{S1,S2}$ on the total volume filled by the sum of the hypervolume of the two niches $V_{S1}$ (species 1) and $V_{S2}$ (species 2). Index D is calculated as follows:

(6) $$D\left(s_{1},s_{2}\right)=\frac{100\times V_{S1,S2}}{V_{S1}+V_{S2}‐V_{S1,S2}}$$

where $V_{S1}=\prod_{i=1}^{p}\beta_{i}$, $V_{S2}=\prod_{i=1}^{p}\gamma_{i},$ and $V_{S1,S2}=\prod_{i=1}^{p}\theta_{i}$, with $\beta_{i}$ and $\gamma_{i}$ the number of category higher or equal to *T* for species 1 and species 2, respectively, and $\theta_{i}$ the number of common categories in the species chromatogram of species 1 and 2, with a joint standardized abundance value (between 0 and 1) higher than or equal to threshold *T*. Only the categories with an abundance > *T* are considered when *T* = 0. *p* is the number of environmental dimensions. When there was no overlap among the two species’ niches, $V_{S1,S2}=0$ and D=0%. When the two species’ niches are identical, $V_{S1}=V_{S2}=V_{S1,S2}$, so D=100%. For this analysis, we assume that the niche has the shape of a *p*‐dimensional orthotope (i.e., the generalization of a rectangle in higher dimensions or hyperrectangle). Therefore, prior to the calculation of index D, we also fill vacant categories between the smallest and the highest selected categories for a given environmental dimension assuming a unimodal (continuous) niche in agreement with ecological niche theory (Brown, 1984; Hutchinson, 1957). We warn that value of D might be biased when the smallest or the highest category (or both) is not well identified. The advantage of our index is that it is not influenced by niche asymmetry.

Index D can be calculated for all species of a taxonomic group and all combinations of dimensions ranging from 1 to *p*. By calculating the average of all values of the matrix, we can identify the most discriminant combinations of environmental dimensions, i.e., the combinations of environmental dimensions that play an important role in term of niche differentiation for the group of species under investigation. Results can be sorted for niches based on a growing number of dimensions from 1 to *p*. (Niche overlapping among species can be assessed by means of the *combina_niche3* function available in the *specieschrom* R package.)

### 2.3 Example with real data

**FIGURE 3:**
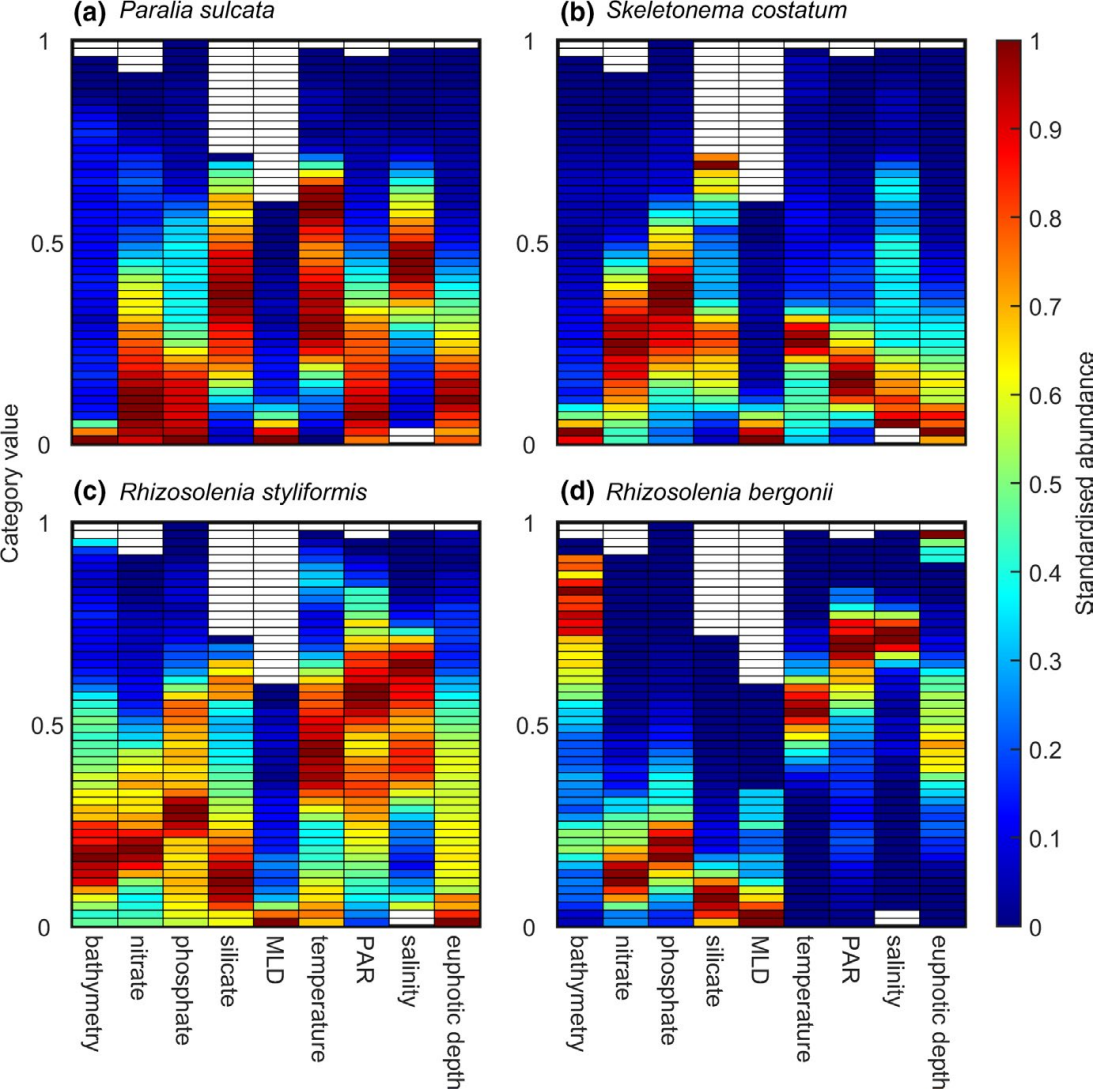
Species chromatograms of four diatom species. Species chromatogram of (a) *Paralia sulcata*, (b) *Skeletonema costatum*, (c) *Rhizosolenia styliformis*, and (d) *Rhizosolenia bergonii*. In a–d, each column represents the species abundance along nine environmental dimensions (i.e., bathymetry, nitrate, phosphate, and silicate concentration, MLD, temperature, PAR, salinity, and euphotic depth). Species abundance in each category (color in the cells) was assessed by estimating the abundance of the 5% of the highest values available in a category if at least 20 CPR samples were available in that category. The Y‐axis corresponds to the 50 categories standardized between 0 and 1. This axis represents all values taken by an environmental variable between 0 and 1 from the lowest (bottom category) to the highest (top category). Colors denote the species abundance standardized between 0 and 1 in each category. High abundance values are in red and low values in blue

**FIGURE 4:**
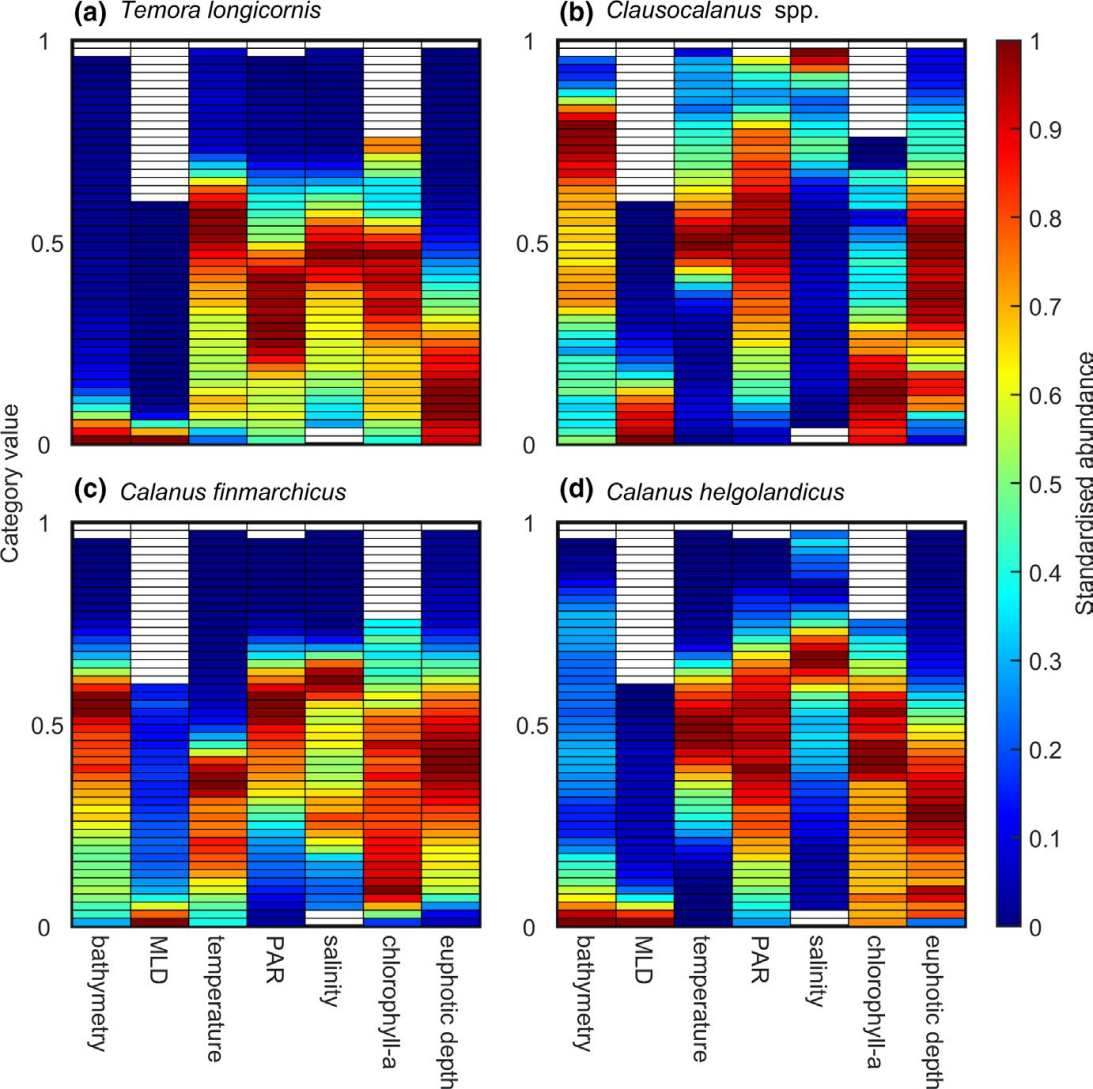
Species chromatograms of four copepods. Species chromatogram of (a) *Temora longicornis*, (b) *Clausocalanus* spp., (c) *Calanus finmarchicus*, and (d) *Calanus helgolandicus*. In a‐d, each column represents the mean species abundance along seven environmental dimensions (i.e., bathymetry, MLD, temperature, PAR, salinity, chlorophyll‐a concentration, and euphotic depth). Species abundance in each category (color in the cells) was assessed by estimating the abundance of the 5% of the highest values available in a category if at least 20 CPR samples were available in that category. The Y‐axis corresponds to the 50 categories standardized between 0 and 1. This axis represents all values taken by an environmental variable between 0 and 1 from the lowest (bottom category) to the highest (top category). Colors denote the species abundance standardized between 0 and 1 in each category. High abundance values are in red and low values in blue

In this study, we used the species chromatogram to display and characterize the multidimensional niche of eight plankton species/taxa into a two‐dimensional space. To do so, we used the abundance data of four diatoms (i.e., *Paralia sulcata*, *Skeletonema costatum*, *Rhizosolenia styliformis*, and *R*. *bergonii*) and four copepods (i.e., *Temora longicornis*, *Clausocalanus* spp., *Calanus finmarchicus*, and *C*. *helgolandicus*) collected by the Continuous Plankton Recorder (CPR) survey between 1998 and 2018 in the North Atlantic Ocean and its adjacent seas (35–65°N and 100°W–10°E). For diatoms, we used nine environmental dimensions (*p* = 9): bathymetry (in m), nitrate, phosphate, and silicate concentrations (mmol m^−3^), Mixed Layer Depth (MLD, m), temperature (°C), Photosynthetically Active Radiations (PAR, E m^−2^ day^−1^), salinity (no unit), and euphotic depth (m) (Figure 3). For copepods, we used seven dimensions (*p* = 7): bathymetry, MLD, temperature, PAR, salinity, chlorophyll‐a concentration, and euphotic depth (Figure 4).

A total of 90,527 CPR samples were used (the repartition of the CPR samples in each category of the chromatograms is displayed in Figure S7). We chose α = 50 categories for each column of the species chromatogram and thresholds of *m* = 1 (as an example to illustrate the method; Figure S1–S6) and *m* = 20 samples (for deep analysis, Figures 3 and 4 and subsequent tables). To handle with the high proportion of nil abundance observed in many CPR samples, we fixed *k* to 5%.

We estimate niche breadth per dimension or average niche breath (all dimensions) and index *D* for the four diatoms and the four copepods by selecting five thresholds: *T* = 0, 0.05, 0.1, 0.25, and 0.5 (Tables 3, 4, 5, 6, S2–S17). Spearman rank correlation coefficients were calculated between niche breadth values obtained for *T* = 0.25 and *T* = 0, 0.05, 0.1, and 0.5. Correlation was tested by means of a Monte Carlo test using 10,000 simulations (Jackson & Somers, 1989).

### 2.4 Comparison with other methods

To test the validity of a method, simulated rather than real data should be used because the former has known distributions and overlaps, whereas the latter might be affected by unknown biases and sampling error (Broennimann et al., 2012). Therefore, to test our approach, we generated seven pseudo‐species (i.e., virtual species) with a three‐dimensional niche using the following equation (Yan & Hunt, 1999):

(7) $$A\left(p_{i}\right)=c\left(\frac{p_{\text{max}}‐p_{i}}{p_{\text{max}}‐p_{\text{opt}}}\right){\left(\frac{p_{i}‐p_{\text{min}}}{p_{\text{opt}}‐p_{\text{min}}}\right)}^{\left(\frac{p_{\text{opt}}‐p_{\text{min}}}{p_{\text{max}}‐p_{\text{opt}}}\right)}$$

with A the abundance of a pseudo‐species along an environmental gradient $p_{i}$, c the maximal abundance (here 1), $p_{\text{opt}}$ the niche optimum along $p_{i},$ and $p_{\text{min}}$ and $p_{\text{max}}$ the amplitude (i.e., niche breadth). Abundances were estimated along three hypothetical environmental dimensions (i.e., $p_{1}$ from 0 to 25, $p_{2}$ from 0 to 40, and $p_{3}$ from 1 to 0). A matrix of 100 samples by three environmental dimensions was obtained and estimated abundances in each sample and along each environmental dimension were aggregated with an additive model. Each pseudo‐species was duplicated (therefore, we considered 7 × 2 = 14 pseudo‐species) to assess the reliability of the overlapping estimation when two niches were identical.

Degrees of niche overlapping between the 14 pseudo‐species were estimated with index *D* (using α = 50, *k* = 5, *m* = 1, and *T* = 0) and with the functions available in the *hypervolume* and *dynRB* R packages using their default parameter settings (Blonder et al., 2018; Junker et al., 2016). Basically, the method developed in the *hypervolume* package uses a hyperellipse random sampling algorithm to generate a uniform random set of points around each observation of the dataset (i.e., a matrix of *n* samples by *p*‐environmental dimensions). Then, a function describing the niche hypervolume is assessed on these points by means of a Gaussian kernel density estimation (Gaussian KDE) or a one‐class Support Vector Machine (one‐class SVM, i.e., an algorithm based on machine learning). The method used in the *dynRB* package is based on an improvement of the concept of multivariate range boxes (Hutchinson, 1957), i.e., a finite number of nested standardized range boxes enclosing a decreasing quantile range of the data are generated and then used to assess a volume and an overlap between each pair of species, the results being subsequently aggregated along all niche dimensions.

**FIGURE 5:**
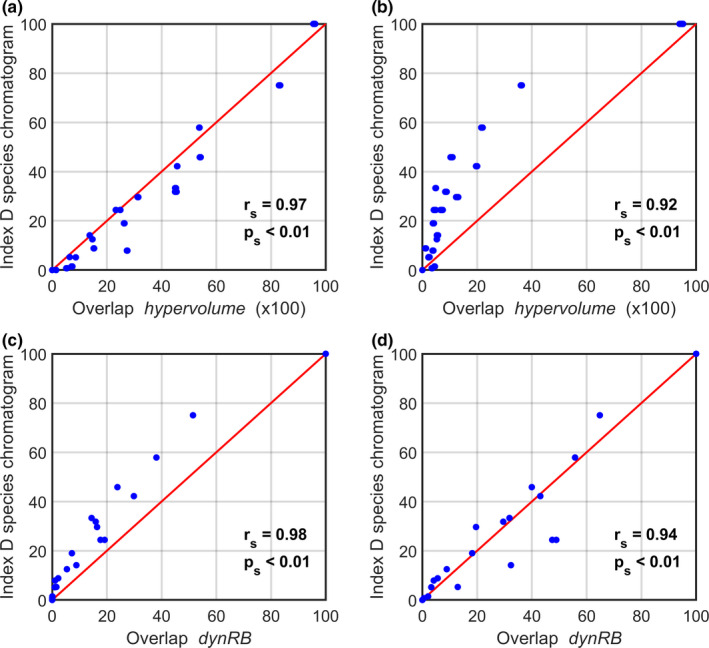
Relationships between the indices of niche overlapping estimated from the species chromatograms (index D) and the indices originating from (a–b) the *hypervolume* (Blonder et al., 2018) and (c–d) the *dynRB* (Junker et al., 2016) R packages. Comparison between niche overlapping of 14 pseudo‐species assessed by means of index *D* and (a–b) the Jaccard similarity coefficient and (c–d) dynamic range boxes. In a and b, pseudo‐species niches hypervolume was delineated with (a) a Gaussian KDE and (b) a one‐class SVM. In c and d, highly correlated environmental dimensions have been (c) kept or (d) replaced with principal components before niche hypervolume estimation. Red line displayed the *y* = *x* relationship. Spearman rank correlation and its associated probability are displayed at the bottom right of each panel

Prior to the estimation with both R packages, the dataset (100 samples by three dimensions) was standardized according to Equation 2. With the *hypervolume* package, niche overlapping was assessed by means of a Jaccard similarity coefficient on niches delineated with (i) a Gaussian KDE (using a Silverman bandwidth estimator; Figure 5a) or (ii) a one‐class SVM (Figure 5b). With the *dynRB* package, niche overlapping was assessed by mean of dynamic range boxes on niches where highly correlated environmental dimensions were (i) kept (Figure 5c) or (ii) replaced with principal components (Figure 5d). As the overlaps estimated with this package are asymmetric (i.e., niche overlap of pseudo‐species $s_{1}$ on pseudo‐species $s_{2}$ is different from the niche overlap of $s_{2}$ on $s_{1}$), we converted the estimations as follow:

(8) $$D_{\text{dynRB}}\left(s_{1},s_{2}\right)=\frac{\left(\text{vol}\left(s_{1}\right)\times \text{port}\left(s_{2},s_{1}\right)\right)\times 100}{\text{vol}\left(s_{1}\right)+\text{vol}\left(s_{2}\right)‐\left(\text{vol}\left(s_{1}\right)\times \text{port}\left(s_{2},s_{1}\right)\right)}$$

With $D_{\text{dynRB}}\left(s_{1},s_{2}\right),$ the symmetric niche overlap between pseudo‐species $s_{1}$ and $s_{2}$, $\text{vol}\left(s_{1}\right)$ the niche volume of pseudo‐species $s_{1}$, $\text{vol}\left(s_{2}\right)$ the niche volume of pseudo‐species $s_{2},$ and $\text{port}\left(s_{2},s_{1}\right)$ the average portion of the niche of $s_{1}$ that is covered by $s_{2}$. We calculated the Spearman rank correlation coefficients between index *D* and the overlaps estimated with the other methods. The correlations were tested by means of a Monte Carlo test using 10,000 permutations (Jackson & Somers, 1989).

## 3 RESULTS

### 3.1 The species chromatograms

Species chromatograms were performed for diatoms and copepods using a threshold *m* = 20 CPR samples to have more reliable estimates of abundance per category (Figures 3 and 4). In this case, the threshold *k* = 5% meant that we performed the average of at least four highest abundance values (0.05 × 20 samples = 4), which decreased the between category variability (Figures 3 and 4 *vs*. Figures S5–S6). The chromatograms allowed a rapid characterization of the niche of these planktonic species. When all environmental dimensions were considered together, all species had a distinct chromatogram (Figures 3 and 4). A visual inspection between patterns exhibited by the species chromatograms (Figures 3 and 4) and patterns in the number of CPR samples (Figure S7) suggests that variation in sampling effort among categories did not influence chromatograms substantially.

Because results of a chromatogram are self‐understandable, we only highlighted a few key patterns. The diatoms *Paralia sulcata* and *Skeletonema costatum*, as well as the copepod *Temora longicornis* and to a lesser extent *Calanus helgolandicus*, were found in shallow regions (i.e., neritic species) in contrast to the diatoms *Rhizosolenia styliformis* and *R*. *bergonii* (oceanic regions) and the copepods *Clausocalanus* spp. and *Calanus finmarchicus* (Figures 3 and 4). *R*. *bergonii* and *Clausocalanus* spp. were thermophilic species, with high abundance toward the top categories for temperature. They also displayed great abundance for high values of PAR, salinity, and euphotic depth. In addition, *R*. *bergonii* showed high abundance for low values of nutrients concentration (Figures 3d and 4b). Their chromatogram therefore suggests that *R*. *bergonii* and *Clausocalanus* spp. are oceanic species and that *R*. *bergonii* is adapted to oligotrophic waters (Figures 3d and 4b).

Some species such as *R*. *styliformis* and *Clausocalanus* spp. displayed large color bands of high abundance along many environmental dimensions (i.e., a color band is an aggregation of more or less continuous categories along an environmental dimension of the chromatogram; Table S1 and Figures 3c and 4b). These large color bands observed along some dimensions revealed large niche breadth with respect to the dimensions. Other species such as *R*. *bergonii* and *T*. *longicornis* exhibited narrower bands of high abundance (e.g., silicate for *R*. *bergonii* and MLD for *T*. *longicornis*) and therefore narrower niche breadth (Figures 3d and 4a).

Some species had complementary chromatograms along some dimensions, e.g., *P*. *sulcata* and *R*. *styliformis* for bathymetry (Figure 3a *vs*. 3c) or *S*. *costatum* and *R*. *bergonii* for temperature, PAR, and salinity (Figure 3b *vs*. 3d). Although two species may have similar chromatograms with respect to some dimensions (e.g., *T*. *longicornis* and *C*. *helgolandicus* along the bathymetric dimensions; Figure 4a and 4d), the same species may be separated by other environmental dimensions (e.g., *T*. *longicornis* and *C*. *helgolandicus* along the dimension salinity; Figure 4a and 4d). Therefore, the species chromatograms can rapidly characterize the full multidimensional complexity of the niche and allow species niche comparisons to be made rapidly.

### 3.2 Estimates of niche optimums

Among diatoms, *P*. *sulcata* had the lowest optimum for bathymetry, PAR, nitrate, and phosphate concentrations but the highest for temperature, suggesting that the diatom was abundant over continental shelves during warm periods (Table 1, Figure 3a). *S*. *costatum* had the highest optimum for nutrients concentration and MLD but the lowest for temperature and salinity, conditions indicative of the spring bloom over cold temperate continental shelves (Caracciolo et al., 2021; Table 1, Figure 3b). The lowest optimum for euphotic depth was found for *R*. *styliformis* (Table 1, Figure 3c). The diatom *R*. *bergonii* had the highest optimum for bathymetry, PAR, salinity, and euphotic depth and the lowest for silicate, some features indicative of oligotrophic waters, which are characteristic of the open ocean during warm‐stratified periods (Table 1, Figure 3d).

**TABLE 1:** Niche optimum assessed from the species chromatogram for each variable and diatom

	*Paralia sulcata*	*Skeletonema costatum*	*Rhizosolenia styliformis*	*Rhizosolenia bergonii*
Bathymetry (m)	43.86	164.87	1024.75	4924.60
Nitrate (mmol m^−3^)	1.53	4.24	3.23	1.86
Phosphate (mmol m^−3^)	0.00	0.40	0.29	0.17
Silicate (mmol m^−3^)	5.24	9.54	1.79	1.26
MLD (m)	18.29	18.34	17.82	17.71
Temperature (°C)	16.45	6.31	11.67	14.66
PAR (E m^−^² day^−1^)	2.34	5.02	19.04	23.03
Salinity (no unit)	33.18	29.48	35.03	35.58
Euphotic depth (m)	20.48	12.97	10.93	102.88

Note

The ecological niche of each species is displayed in Figure 3.

Among zooplankton, *T*. *longicornis* had the lowest niche optimum for bathymetry, MLD, PAR, salinity, and euphotic depth but the highest for temperature and chlorophyll‐a concentration (Table 2, Figure 4a). The lowest thermal optimum was found for *C*. *finmarchicus*, a subarctic oceanic species, which also had the highest optimum for PAR and the lowest for chlorophyll‐a concentration (Table 2 and Figure 4c). A low optimum was also observed with respect to chlorophyll‐a concentration for *Clausocalanus* spp., a warm temperate oceanic genus. This copepod had optimums for higher values of bathymetry, MLD, salinity, and euphotic depth (Table 2 and Figure 4b). *C*. *helgolandicus* had intermediate optimums for all the variables (Table 2 and Figure 4d).

**TABLE 2:** Niche optimum assessed from the species chromatogram for each variable and copepod

	*Temora longicornis*	*Clausocalanus* spp.	*Calanus finmarchicus*	*Calanus helgolandicus*
Bathymetry (m)	45.01	4716.65	3166.20	81.85
MLD (m)	15.60	18.27	17.57	16.84
Temperature (°C)	15.85	13.47	9.28	13.46
PAR (E m^−^² day^−1^)	9.70	17.70	18.36	13.01
Salinity (no unit)	33.37	38.01	34.66	35.21
Chlorophyll‐a (mg m^−3^)	2.08	0.59	0.41	1.83
Euphotic depth (m)	18.25	58.92	51.31	38.22

Note

The ecological niche of each species/taxa is displayed in Figure 4.

### 3.3 Estimates of niche breadth

Niche breadth was assessed for each species based on five different thresholds of abundance *T*. Only results for *T* = 0.25 (Tables 3 and 4) are described in detail here; results with other thresholds are shown in Tables S2–S9. Among diatoms, *R*. *styliformis* had the highest average niche breadth (*E_T_* = 73.02%; Table 3 and Figure 3c). Among studied copepods, *Clausocalanus* spp. was the most euryoecious (average niche breadth *E_T_* = 68.42%; Table 4 and Figure 4b). In contrast, *R*. *bergonii* and *T*. *longicornis* had the narrowest average niche breadth, with *E_T_* = 42.21% and *E_T_* = 53.36%, respectively (Tables 3 and 4 and Figures 3d and 4a). Niche breadth sometimes exhibited very different values among diatoms or copepods for the same dimensions. For example, although niche breadth for bathymetry was ~6% for *P*. *sulcata* and 12.5% for *T*. *longicornis*, niche breadth was 100% and ~92% for *R*. *styliformis* and *Clausocalanus* spp., respectively (Tables 3, 4 and Figures 3a, 4a, 3c, and 4b). Within a species, e.g., *R*. *bergonii*, large niche breadth (e.g., bathymetry) could be found for a niche dimension, whereas narrow niche breadth (e.g., salinity) could be observed for another (Table 3 and Figure 3d). Among copepods, *C*. *helgolandicus* had the narrowest niche for temperature but one of the largest for PAR (Table 4 and Figure 4d).

**TABLE 3:** Niche breadth (ecological niche breadth) assessed from the species chromatogram of the four diatoms based on a threshold of abundance *T* = 0.25

	*Paralia sulcata*	*Skeletonema costatum*	*Rhizosolenia styliformis*	*Rhizosolenia bergonii*
Bathymetry (%)	6.25	10.42	100.00	81.25
Nitrate (%)	54.35	52.17	54.35	34.78
Phosphate (%)	58.00	58.00	64.00	36.00
Silicate (%)	86.11	88.89	97.22	25.00
MLD (%)	16.67	16.67	26.67	56.67
Temperature (%)	59.18	34.69	91.84	26.53
PAR (%)	50.00	31.25	85.42	35.42
Salinity (%)	48.94	68.09	74.47	14.89
Euphotic depth (%)	44.90	34.69	63.27	69.39
***E***_***T***_ **(%)**	**47.15**	**43.87**	**73.02**	**42.21**

Note

The mean niche breadth ($E_{T}$) for all dimensions and each species is also displayed in bold. The ecological niche of each diatom is shown in Figure 3.

**TABLE 4:** Niche breadth (ecological niche breadth) assessed from the species chromatogram of the four copepods based on a threshold of abundance *T* = 0.25

	*Temora longicornis*	*Clausocalanus spp*.	*Calanus finmarchicus*	*Calanus helgolandicus*
Bathymetry (%)	12.50	91.67	70.83	83.33
MLD (%)	10.00	33.33	20.00	13.33
Temperature (%)	69.39	59.18	48.98	44.90
PAR (%)	68.75	91.67	54.17	79.17
Salinity (%)	65.96	34.04	61.70	61.70
Chlorophyll‐a (%)	100.00	89.47	97.37	94.74
Euphotic depth (%)	46.94	79.59	65.31	59.18
***E***_***T***_ **(%)**	**53.36**	**68.42**	**59.77**	**62.34**

Note

The mean niche breadth ($E_{T}$) for all dimensions and each species/taxa is also displayed in bold. The ecological niche of each copepod is shown in Figure 4.

Altering threshold *T* did affect estimates of niche breadth but this effect was small when the modification on *T* was moderate (Tables S2–S9). For diatoms, Spearman rank correlation between niche breadth based on *T* = 0.25 and *T* = 0, 0.05, 0.1, and 0.5 was 0.45 (degree of freedom df = 36, *p* < .05), 0.68 (df = 36, *p* < .01), 0.82 (df = 36, *p* < .01), and 0.88 (df = 36, *p* < .01), respectively. For copepods, Spearman rank correlation between niche breadth based on *T* = 0.25 and *T* = 0, 0.05, 0.1, and 0.5 was 0.69 (df = 28, *p* < .01), 0.69 (df = 28, *p* < .01), 0.84 (df = 28, *p* < .01), and 0.85 (df = 28, *p* < .01), respectively.

### 3.4 Niche differentiation

Finally, we investigated which sets of environmental variables were the most discriminant in term of niche differentiation among species/taxa of the same taxonomic group. As for niche breadth, the degree of niche overlapping D was assessed for five different thresholds of abundance *T*. Results for *T* = 0.25 are shown in detail here (Tables 5 and 6) and results based on other thresholds are shown in Tables S10–S17.

**TABLE 5:** Mean degree of niche overlapping for the four diatoms based on a threshold of abundance *T* = 0.25

Number of dimensions	Combinations									Index *D* (%)
1	1									26.32
2	1	6								9.20
3	1	6	8							4.79
4	1	6	7	8						3.15
5	1	6	7	8	9					2.49
6	1	4	6	7	8	9				2.34
7	1	4	5	6	7	8	9			2.24
8	1	2	4	5	6	7	8	9		2.17
9	1	2	3	4	5	6	7	8	9	2.14

Note

The first column displays the number of dimensions considered simultaneously, columns 2 to 10 display the combinations of dimensions (i.e., 1 = bathymetry, 2 = nitrate, 3 = phosphate, 4 = silicate, 5 = MLD, 6 = temperature, 7 = PAR, 8 = salinity, and 9 = euphotic depth). The last column displays index *D* associated with the combination of environmental dimensions. *D* = 0% when species niches are different and *D* = 100% when species niches are identical; the higher the number of dimensions, the lower the value of index *D*. Only the combinations of environmental variables that minimize values of index *D* are displayed. The ecological niche of each species is displayed in Figure 3.

**TABLE 6:** Mean degree of niche overlapping for the four copepods based on a threshold of abundance *T* = 0.25

Number of dimensions	Combinations							Index *D* (%)
1	5							34.17
2	1	5						18.05
3	1	3	5					10.05
4	1	2	3	5				6.38
5	1	2	3	5	7			5.01
6	1	2	3	4	5	7		4.17
7	1	2	3	4	5	6	7	4.07

Note

The first column displays the number of dimensions considered simultaneously, columns 2 to 8 display the combinations of dimensions (i.e., 1 = bathymetry, 2 = MLD, 3 = temperature, 4 = PAR, 5 = salinity, 6 = chlorophyll‐a concentration, and 7 = euphotic depth) and the last column displays index *D* associated with the combination of environmental dimensions. *D* = 0% when species niches are different and *D* = 100% when species niches are identical; the higher the number of dimensions, the lower the value of index *D*. Only the combinations of environmental variables that minimize values of index *D* are displayed. The ecological niche of each copepod is shown in Figure 4.

When only one environmental dimension was considered, the most discriminant variable, i.e., the variable that allowed niche overlapping to be the smallest was, for diatoms (for *T* = 0.25), bathymetry (D = 26.32%), followed by PAR (32.97%), temperature (34.56%), euphotic depth (42.35%), salinity (42.51%), silicate (54.24%), MLD (55.15%), phosphate (76.94%), and finally nitrate (81.11%). For zooplankton species/taxa, the most discriminant variable was salinity (D = 34.17%), followed by temperature (42.02%), bathymetry (49.91%), MLD (53.61%), euphotic depth (68.49%), PAR (69.03%), and chlorophyll‐a (92.5%).

We then examined the effect of the combinations of environmental variables on index D. Expectedly, for diatoms or copepods, we found that the lowest mean degree of niche overlapping was reached when the number of niche dimensions considered was highest (Tables 5 and 6 and Tables S10–S17). For diatoms, when only two environmental variables were considered, bathymetry and temperature was the combination of variables that allowed to best separate their niches (i.e., lowest degree of niche overlapping, Table 5). For zooplankton species/taxa, this was bathymetry and salinity (Table 6). For diatoms, when four environmental variables were considered, the lowest value of D was found with the combination of variables bathymetry, temperature, PAR, and salinity (Table 5). For zooplankton, the lowest value of D was reached for the combination of bathymetry, MLD, temperature and salinity (Table 6).

The use of other thresholds *T* could lead to the detection of other combinations of variables but in general there was a high consistency in the combinations from *T* = 0 to *T* = 0.5 (Tables 5 and 6, S10–S17).

### 3.5 Comparison of species chromatograms with other methods

The three‐dimensional niches of 14 pseudo‐species were examined by means of (i) species chromatograms and (ii) sets of pair plots from the *hypervolume* package (Blonder et al., 2018). A visual comparison of the figures revealed that both procedures gave similar results (Figures S8 vs. S9–S10). In our hypothetical examples, both methods easily identified which pseudo‐species had overlapping or non‐overlapping niches (e.g., pseudo‐species 8 *vs*. 11 and 7 v*s*. 14, respectively) and enabled the comparison of the niche breadth of all pseudo‐species along all niche dimensions (e.g., pseudo‐species 5 had a larger niche breadth than pseudo‐species 4). However, because the procedure used in the *hypervolume* method does not consider abundance, optimums cannot be visually identified which can make interpretations difficult. For example, according to the *hypervolume* display (Figures S9–S10), pseudo‐species 1 and 9 seemed to have very similar niches but the species chromatogram display revealed that they had different niche optimums (Figure S8).

We also compared the degree of niche overlapping estimated from our index *D* with the *hypervolume* or *dynRB* packages (Figure 5). Although the relationships were not always linear (Figure 5b,c), we found comparable degree of niche overlapping with both methods (Spearman correlation *r_s_* > 0.9 and *p_s_* < 0.01), even when the procedures used to delineate the niche were different (Figure 5a *vs*. 5b) or when the dimensionality was reduced by means of a PCA (Figure 5c *vs*. 5d). However, we noticed that niche overlapping estimated by means of the *hypervolume* method never reached 100%, even when both pseudo‐species had the same niche (Figures 5a,b and S8), an issue that has already been reported elsewhere (Junker et al., 2016).

## 4 DISCUSSION

The chromatography is a physicochemical method used to separate the different components of a mixture. This mixture, dissolved in a fluid, is allowed to travel in a system including a fixed stationary phase. The mixture migrates along papers or polymers at a velocity that depends upon the characteristics of the molecules, which enable them to be separated. Some methods show the results under the form of a diagram, called a chromatogram, with different colored bands, each reflecting a different component of the fluid. (We recall that the Greek etymology of the word “chromatography” means “to write in colour.”) Although very different, our procedure leads to a graphic that can be called a species chromatogram, by analogy with the classical physico‐chemical method. In a species chromatogram, colored bands spread along different environmental gradients for each dimension of the niche.

The species chromatogram method summarizes rapidly the niche of a species and enables rapid comparisons to be made. Comparison between chromatograms is possible because of the double standardization between 0 and 1, i.e., (i) the standardization of each environmental dimension considering all species involved in a study (a continuous and unitless environmental dimension is an essential prerequisite for comparing different hypervolumes in an Euclidean space (Blonder, 2018)) and (ii) the standardization of the abundance for all categories of a given environmental dimension. From a chromatogram, one can identify niche optimums and breaths with respect to all niche dimensions. A rapid quantification of the difference among niches can also be undertaken, which is important to evaluate the degree of niche overlapping among species. Least and not last, the method allows combinations of environmental dimensions that minimize niche overlapping to be identified.

Other methods have been proposed to represent the niche of a species. Among them, the simplest and most efficient is perhaps the one used in the *hypervolume* R package (Blonder et al., 2014), which consists in a set of pair plots for all dimensions that define the space where the hypervolume belongs to. However, as $p\times \left(p‐1\right)/2$ combinations of dimensions are possible and because each variable is represented according to another, it would have led here for a single species to 21 figures when *p* = 7 and 36 when *p* = 9. Ordination methods can also be applied to characterize the ecological niche of a species (Dolédec et al., 2000; Ter Braak & Prentice, 1988). Among them, a principal component analysis (PCA) has already been used to represent the niche of *C*. *finmarchicus* and *C*. *helgolandicus* based on more than 10 environmental variables in the North Atlantic Ocean (Helaouët & Beaugrand, 2007). Principal components (PCs) enabled the reduction in the number of environmental dimensions, and species abundance was then represented in a space defined by the different PCs (three in Helaouët & Beaugrand, 2007). However, PCs are linear combinations of environmental factors, and therefore the resulting assessment of the multidimensional niche is difficult to interpret because the weight of each environmental dimension in the PCs is not so easy to understand and some variables can be represented in more than one PC. Furthermore, as in many multivariate techniques applied at a species or a community level, the PCs may sometimes explain a small fraction of the variance (Ter Braak & Prentice, 1988).

In contrast, a species chromatogram enables a simple characterization of the niche of a species and allows niches to be compared. Multidimensional niches are not summarized by creating composite variables and all the environmental dimensions are used (even if correlated) to display a species niche. Therefore, niche holes (i.e., unoccupied part of the niche which are difficult to detect but indicative of important ecological and evolutionary processes (Blonder, 2016)) can be easily identified. Differences and similarities among niches can be visually assessed, allowing the examination of three ecological phenomena: (i) Hutchinson's duality (Colwell & Rangel, 2009), (ii) environmental filtering (Zobel, 1997) and (iii) niche complementarity (i.e., the niche differentiation effect) (Tilman, 1999). Last and not least, the role and contribution of each environmental dimension to a species niche can be easily assessed.

Our method has some limitations. First, the value of threshold *T* influences the estimation of the niche breadth and the degree of niche overlapping among species and different results can be observed for different thresholds (Tables 3 and 6, S2–S17). Although a few differences were found, results remained quite consistent especially for copepods. Estimating the degree of overlapping and niche breadth is difficult especially in the pelagic environment because of the absence of strong physical barriers (van der Spoel, 1994). Therefore, the realized niche can be larger than the fundamental niche because of species dispersal (also called species expatriation) (Pulliam, 2000). The application of our numerical procedures on terrestrial data may show less variability for different values of *T*.

Second, large variability in the abundance estimates of each category of the chromatogram can also occur (Figure S1). This large variability has two main causes. The first cause is related to the fact that the abundance of a species within a category of a given environmental dimension is also influenced by the range of conditions that also occurs in other dimensions. Having a nil abundance in a category corresponding to optimal conditions for a particular dimension is possible when other dimensions have unsuitable environmental conditions. That is why we calculated the average abundance corresponding to the *k* % of the highest values available within a given category. The second cause is more inherent to the CPR survey. The CPR machine samples ~3 m^3^ of seawater but the range of filtered water can vary between 2 and 5 m^3^ depending on ship speed and plankton concentration in the water column (Jonas et al., 2004). Variation in seawater filtered may have severe consequences for abundance estimation.

Third, empty (white) categories were observed for some dimensions of the chromatogram, e.g., silicate, MLD, or euphotic depth (Figures 3 and 4). These empty categories were due to an insufficient number of CPR samples and was reduced when threshold *m* diminished from 20 to 1 sample(s), although outliers appeared at the same time (Figures S5 and S6 *vs*. Figures 3 and 4). However, empty categories could still be observed along some dimensions because some environmental conditions are rarely observed in the North Atlantic sector, i.e., fundamental environmental conditions are not always realized (Jackson & Overpeck, 2000). The smoothing of the data also exacerbated the number of missing categories by altering the location of some white categories in the chromatograms (see the chlorophyll‐a dimension in Figures S2 vs. S4). Adjusting the order (i.e., degree of smoothing) of the simple moving average may be necessary if the method is applied to other datasets.

Last, we assimilated the shape of the multidimensional niche to a hyperrectangle in order to estimate the degree of niche overlapping (index *D*). This assumption agrees with the definition of the niche *sensu* Hutchinson (1957), which supposed an equal probability of persistence in each point composing the fundamental niche, even if suboptimal conditions should be observed near the boundaries. More complicated shapes are observed with the realized niche because of the distortions and the modulations generated by biotic interactions that create unoccupied spaces, e.g., niche holes (Blonder, 2016; Soberón & Peterson, 2020). Therefore, prior to overlapping estimation, we filled the unoccupied categories along each dimension, which assimilated niche shape to a hyperrectangle, an assumption that was tested through a comparison we performed with the *hypervolume* and *dynRB* packages (Figure 5).

During the last decade, many approaches have been developed to characterize a niche hypervolume, each with their own assumptions and drawbacks (Blonder, 2018; Qiao et al., 2015, 2017; Soberon & Nakamura, 2009). However, some methods are more easily applicable than other. For example, the *dynRB* package has been developed to provide reliable results using default parameter settings (Junker et al., 2016). Although it has been recently updated, the *hypervolume* method requires expert knowledge and might therefore be misused because of the numerous assumptions underlying its application (Blonder et al., 2017, 2018; Qiao et al., 2017). Furthermore, as the method uses an algorithm which generates a random set of points, it cannot assess total niche overlapping (i.e., an overlapping of 100%) between two pseudo‐species having the same niche (Figure 5a,b). In contrast, a species chromatogram is simple and easily understandable. The method does not require the selection of multiple thresholds and underlying functions. Our technique is, therefore, reproducible by a broad range of ecologists and might be straightforwardly adaptable to various datasets and conditions.

## 5 CONCLUSIONS

The species chromatogram is a simple and visually appealing method enabling a clear and rapid representation of the (multidimensional) ecological niche of a species into a two‐dimensional space. The method thereby allows one to characterize the full multidimensional complexity of the niche of a species. The niche is displayed with *p*‐standardized gradients figuring the continuous variation in each environmental variable, defining the niche axes from the lowest to the highest value taken by each variable, each gradient being divided into α equidistant categories filled with species’ abundance. A species chromatogram can be seen as a species’ fingerprint, summarizing its environmental requirements. Although we only used quantitative variables, semi‐quantitative or qualitative variables can also be selected. A precise estimation of niche optimums and breadths along each dimension is also possible from a chromatogram. In addition, the quantification of the degree of niche overlapping can be made, which rapidly identifies the most discriminant combinations of environmental variables that minimize niche overlapping among species. Although we applied the method on marine plankton species, the species chromatogram can also be applied to terrestrial data.

## CONFLICT OF INTEREST

The authors declare no conflict of interest.

## AUTHOR CONTRIBUTIONS

**Loïck Kléparski:** Conceptualization (equal); Data curation (equal); Formal analysis (equal); Investigation (lead); Methodology (equal); Software (equal); Validation (lead); Visualization (lead); Writing – original draft (lead); Writing – review & editing (lead). **Grégory Beaugrand:** Conceptualization (equal); Data curation (equal); Formal analysis (equal); Investigation (supporting); Methodology (equal); Software (equal); Supervision (lead); Validation (supporting); Visualization (supporting); Writing – original draft (supporting); Writing – review & editing (supporting).

## Supporting information

Appendix S1

## Data Availability

All data used in this study are freely available (see the *Materials and Methods* section). The full method has been implemented in a R package (*specieschrom*) available on Github (https://github.com/loick‐klpr/specieschrom.git) and as Matlab functions (https://github.com/loick‐klpr/Species‐chromatogram‐with‐Matlab.git).
