# Supplementary material for: The species chromatogram, a new graphical method to represent, characterize, and compare the ecological niches of different species
Source: Ecol Evol. 2022 Apr 13;12(4):e8830. doi: 10.1002/ece3.8830 (PMC9006236; doi:10.1002/ece3.8830)
Supplement: Supplementary file 1 — Appendix S1 [file ECE3-12-e8830-s001.docx]

**The species chromatogram, a new graphical method to represent, characterise and compare the ecological niches of different species**

Loïck Kléparski ^1,2 *^, Grégory Beaugrand ^1^

^1^ Univ. Littoral Côte d’Opale, CNRS, Univ. Lille, UMR 8187 - LOG - Laboratoire d’Océanologie et de Géosciences, F-62930 Wimereux, France

^2^ Marine Biological Association, Citadel Hill, Plymouth PL1 2PB, United Kingdom.

**Supplementary Tables.**

**Table S1. Glossary of the technical terms used in this paper.**

| **Term** | **Definition** | **Reference** |
| --- | --- | --- |
| **Ecological niche** | Although different definitions of the niche have been proposed, the concept is here defined as the set of environmental conditions allowing a species to growth, maintain and reproduce. The niche is therefore assimilated to a p-dimensional hypervolume (with p corresponding to the number of environmental variables) in which each environmental combination enables a species to exist indefinitely (i.e. the species fundamental niche). | (Hutchinson, 1957, 1978) |
| **Community chromatogram** | A graphic, originally called environmental chromatogram, that displays where species of an assemblage aggregate along multiple environmental dimensions. | (Kléparski et al., 2021) |
| **Species chromatogram** | A graphic that displays the (multidimensional) ecological niche of a species into a two-dimensional space. | - |
| **Environmental signature** | The set of environmental conditions where a species assemblage (or a community) is typically found. | - |
| **Niche optimum** | The average environmental value where a species has its highest abundance along one niche dimension. | - |
| **Niche breadth** | A measure of the environmental range where a species is abundant. In our study, niche breadth was assessed by the number of categories of the species chromatogram where abundance was higher or equal to a threshold T along an environmental gradient, with 0<T≤1. Five values of T were tested: T=0, T=0.05, T=0.1, T=0.25 and T=0.5. | - |
| **Average niche breadth** | The average of niche breadth based on all environmental dimensions of the species chromatogram. | - |
| **Degree of niche overlapping** | The percentage of the niche hypervolume common to different species. | - |
| **Eury/stenography** | Terms used to characterise the spatial distribution of a species. A species with a large spatial distribution is eurygraphic whereas a species with a narrow spatial distribution is stenographic. | - |
| **Environmental gradient** | This term refers to the continuous variation of an environmental variable from its lowest to its highest value. | - |
| **Environmental variable** | The state of a biotic (e.g. chlorophyll-a concentration) or abiotic (e.g. sea water temperature) parameter that can be quantitative or qualitive. Only quantitative variables were used in this study. | - |
| **Environmental dimension** | An axis of the niche defined by an environmental variable. | - |
| **Coloured band** | In a community chromatogram, an aggregation of more or less continuous categories with a significant percentage of aggregation. In a species chromatogram, more or less continuous categories with an abundance above a threshold along an environmental dimension. | - |

**Table S2. Niche breadth (ecological niche breadth) assessed from the species chromatogram of the four diatoms based on a threshold of abundance T=0.** The mean niche breadth ($E_{T}$) for all dimensions and each species is also displayed in bold. The ecological niche of each diatom is shown in Figure 3.

|  | ***Paralia sulcata*** | ***Skeletonema costatum*** | ***Rhizosolenia styliformis*** | ***Rhizosolenia bergonii*** |
| --- | --- | --- | --- | --- |
| **Bathymetry (%)** | 100.00 | 95.83 | 100.00 | 95.83 |
| **Nitrate (%)** | 91.30 | 84.78 | 100.00 | 89.13 |
| **Phosphate (%)** | 92.00 | 92.00 | 100.00 | 94.00 |
| **Silicate (%)** | 100.00 | 100.00 | 97.22 | 80.56 |
| **MLD (%)** | 86.67 | 83.33 | 93.33 | 60.00 |
| **Temperature (%)** | 97.96 | 97.96 | 97.96 | 85.71 |
| **PAR (%)** | 91.67 | 87.50 | 95.83 | 89.58 |
| **Salinity (%)** | 100.00 | 93.62 | 87.23 | 51.06 |
| **Euphotic depth (%)** | 100.00 | 87.76 | 100.00 | 89.80 |
| **E_T_ (%)** | **95.51** | **91.42** | **96.84** | **81.74** |

**Table S3. Niche breadth (ecological niche breadth) assessed from the species chromatogram of the four diatoms based on a threshold of abundance T=0.05.** The mean niche breadth ($E_{T}$) for all dimensions and each species is also displayed in bold. The ecological niche of each diatom is shown in Figure 3.

|  | ***Paralia sulcata*** | ***Skeletonema costatum*** | ***Rhizosolenia styliformis*** | ***Rhizosolenia bergonii*** |
| --- | --- | --- | --- | --- |
| **Bathymetry (%)** | 87.50 | 72.92 | 100.00 | 95.83 |
| **Nitrate (%)** | 84.78 | 76.09 | 84.78 | 52.17 |
| **Phosphate (%)** | 68.00 | 78.00 | 90.00 | 56.00 |
| **Silicate (%)** | 97.22 | 100.00 | 97.22 | 44.44 |
| **MLD (%)** | 60.00 | 66.67 | 90.00 | 56.67 |
| **Temperature (%)** | 81.63 | 97.96 | 97.96 | 40.82 |
| **PAR (%)** | 75.00 | 62.50 | 95.83 | 77.08 |
| **Salinity (%)** | 74.47 | 89.36 | 80.85 | 42.55 |
| **Euphotic depth (%)** | 59.18 | 51.02 | 100.00 | 83.67 |
| **E_T_ (%)** | **76.42** | **77.17** | **92.96** | **61.03** |

**Table S4. Niche breadth (ecological niche breadth) assessed from the species chromatogram of the four diatoms based on a threshold of abundance T=0.1.** The mean niche breadth ($E_{T}$) for all dimensions and each species is also displayed in bold. The ecological niche of each diatom is shown in Figure 3.

|  | ***Paralia sulcata*** | ***Skeletonema costatum*** | ***Rhizosolenia styliformis*** | ***Rhizosolenia bergonii*** |
| --- | --- | --- | --- | --- |
| **Bathymetry (%)** | 85.42 | 35.42 | 100.00 | 91.67 |
| **Nitrate (%)** | 80.43 | 56.52 | 67.39 | 43.48 |
| **Phosphate (%)** | 62.00 | 62.00 | 72.00 | 50.00 |
| **Silicate (%)** | 91.67 | 94.44 | 97.22 | 33.33 |
| **MLD (%)** | 40.00 | 23.33 | 53.33 | 56.67 |
| **Temperature (%)** | 69.39 | 63.27 | 95.92 | 32.65 |
| **PAR (%)** | 62.50 | 52.08 | 93.75 | 72.92 |
| **Salinity (%)** | 72.34 | 72.34 | 78.72 | 19.15 |
| **Euphotic depth (%)** | 53.06 | 44.90 | 81.63 | 83.67 |
| **E_T_ (%)** | **68.53** | **56.03** | **82.22** | **53.73** |

**Table S5. Niche breadth (ecological niche breadth) assessed from the species chromatogram of the four diatoms based on a threshold of abundance T=0.5.** The mean niche breadth ($E_{T}$) for all dimensions and each species is also displayed in bold. The ecological niche of each diatom is shown in Figure 3.

|  | ***Paralia sulcata*** | ***Skeletonema costatum*** | ***Rhizosolenia styliformis*** | ***Rhizosolenia bergonii*** |
| --- | --- | --- | --- | --- |
| **Bathymetry (%)** | 4.17 | 6.25 | 37.50 | 77.08 |
| **Nitrate (%)** | 45.65 | 39.13 | 36.96 | 21.74 |
| **Phosphate (%)** | 26.00 | 40.00 | 60.00 | 16.00 |
| **Silicate (%)** | 72.22 | 83.33 | 91.67 | 19.44 |
| **MLD (%)** | 10.00 | 13.33 | 10.00 | 16.67 |
| **Temperature (%)** | 51.02 | 12.24 | 65.31 | 16.33 |
| **PAR (%)** | 37.50 | 20.83 | 62.50 | 25.00 |
| **Salinity (%)** | 34.04 | 19.15 | 72.34 | 12.77 |
| **Euphotic depth (%)** | 32.65 | 20.41 | 51.02 | 61.22 |
| **E_T_ (%)** | **34.81** | **28.30** | **54.14** | **29.58** |

**Table S6. Niche breadth (ecological niche breadth) assessed from the species chromatogram of the four copepods based on a threshold of abundance T=0.** The mean niche breadth ($E_{T}$) for all dimensions and each species/taxa is also displayed in bold. The ecological niche of each copepod is shown in Figure 4.

|  | ***Temora longicornis*** | ***Clausocalanus spp.*** | ***Calanus finmarchicus*** | ***Calanus helgolandicus*** |
| --- | --- | --- | --- | --- |
| **Bathymetry (%)** | 97.92 | 100.00 | 97.92 | 97.92 |
| **MLD (%)** | 100.00 | 93.33 | 100.00 | 93.33 |
| **Temperature (%)** | 100.00 | 100.00 | 100.00 | 95.92 |
| **PAR (%)** | 97.92 | 100.00 | 100.00 | 100.00 |
| **Salinity (%)** | 100.00 | 97.87 | 89.36 | 100.00 |
| **Chlorophyll-a (%)** | 100.00 | 100.00 | 100.00 | 100.00 |
| **Euphotic depth (%)** | 100.00 | 100.00 | 100.00 | 100.00 |
| **E_T_ (%)** | **99.40** | **98.74** | **98.18** | **98.17** |

**Table S7. Niche breadth (ecological niche breadth) assessed from the species chromatogram of the four copepods based on a threshold of abundance T=0.05.** The mean niche breadth ($E_{T}$) for all dimensions and each species/taxa is also displayed in bold. The ecological niche of each copepod is shown in Figure 4.

|  | ***Temora longicornis*** | ***Clausocalanus spp.*** | ***Calanus finmarchicus*** | ***Calanus helgolandicus*** |
| --- | --- | --- | --- | --- |
| **Bathymetry (%)** | 33.33 | 100.00 | 79.17 | 89.58 |
| **MLD (%)** | 13.33 | 50.00 | 100.00 | 76.67 |
| **Temperature (%)** | 100.00 | 95.92 | 59.18 | 59.18 |
| **PAR (%)** | 75.00 | 100.00 | 72.92 | 87.50 |
| **Salinity (%)** | 72.34 | 91.49 | 72.34 | 97.87 |
| **Chlorophyll-a (%)** | 100.00 | 89.47 | 100.00 | 100.00 |
| **Euphotic depth (%)** | 59.18 | 100.00 | 85.71 | 73.47 |
| **E_T_ (%)** | **64.74** | **89.55** | **81.33** | **83.47** |

**Table S8. Niche breadth (ecological niche breadth) assessed from the species chromatogram of the four copepods based on a threshold of abundance T=0.1.** The mean niche breadth ($E_{T}$) for all dimensions and each species/taxa is also displayed in bold. The ecological niche of each copepod is shown in Figure 4.

|  | ***Temora longicornis*** | ***Clausocalanus spp.*** | ***Calanus finmarchicus*** | ***Calanus helgolandicus*** |
| --- | --- | --- | --- | --- |
| **Bathymetry (%)** | 20.83 | 100.00 | 77.08 | 89.58 |
| **MLD (%)** | 13.33 | 43.33 | 100.00 | 23.33 |
| **Temperature (%)** | 73.47 | 65.31 | 53.06 | 53.06 |
| **PAR (%)** | 72.92 | 95.83 | 68.75 | 87.50 |
| **Salinity (%)** | 70.21 | 38.30 | 72.34 | 80.85 |
| **Chlorophyll-a (%)** | 100.00 | 89.47 | 100.00 | 100.00 |
| **Euphotic depth (%)** | 53.06 | 100.00 | 73.47 | 69.39 |
| **E_T_ (%)** | **57.69** | **76.03** | **77.81** | **71.96** |

**Table S9. Niche breadth (ecological niche breadth) assessed from the species chromatogram of the four copepods based on a threshold of abundance T=0.5.** The mean niche breadth ($E_{T}$) for all dimensions and each species/taxa is also displayed in bold. The ecological niche of each copepod is shown in Figure 4.

|  | ***Temora longicornis*** | ***Clausocalanus spp.*** | ***Calanus finmarchicus*** | ***Calanus helgolandicus*** |
| --- | --- | --- | --- | --- |
| **Bathymetry (%)** | 8.33 | 89.58 | 60.42 | 10.42 |
| **MLD (%)** | 6.67 | 26.67 | 10.00 | 10.00 |
| **Temperature (%)** | 63.27 | 26.53 | 38.78 | 32.65 |
| **PAR (%)** | 54.17 | 81.25 | 37.50 | 62.50 |
| **Salinity (%)** | 48.94 | 6.38 | 51.06 | 17.02 |
| **Chlorophyll-a (%)** | 94.74 | 42.11 | 73.68 | 86.84 |
| **Euphotic depth (%)** | 34.69 | 63.27 | 55.10 | 51.02 |
| **E_T_ (%)** | **44.40** | **47.97** | **46.65** | **38.64** |

**Table S10. Mean degree of niche overlapping for the four diatoms based on a threshold of abundance T=0.** The first column displays the number of dimensions considered simultaneously, columns 2 to 10 display the combinations of dimensions (i.e. 1=bathymetry, 2=nitrate, 3=phosphate, 4=silicate, 5=MLD, 6=temperature, 7=PAR, 8=salinity and 9=euphotic depth). The last column displays index D associated with the combination of environmental dimensions. D=0% when species niches are different and D=100% when species niches are identical; the higher the number of dimensions, the lower the value of index D. Only the combinations of environmental variables that minimise values of index D are displayed. The ecological niche of each species is displayed in Figure 3.

| **Number of dimensions** | **Combinations** | | | | | | | | | **Index D (%)** |
| --- | --- | --- | --- | --- | --- | --- | --- | --- | --- | --- |
| **1** | 8 |  |  |  |  |  |  |  |  | 73.03 |
| **2** | 5 | 8 |  |  |  |  |  |  |  | 61.31 |
| **3** | 5 | 8 | 9 |  |  |  |  |  |  | 55.55 |
| **4** | 2 | 5 | 8 | 9 |  |  |  |  |  | 50.92 |
| **5** | 2 | 4 | 5 | 8 | 9 |  |  |  |  | 47.56 |
| **6** | 2 | 4 | 5 | 7 | 8 | 9 |  |  |  | 45.29 |
| **7** | 2 | 3 | 4 | 5 | 7 | 8 | 9 |  |  | 43.46 |
| **8** | 2 | 3 | 4 | 5 | 6 | 7 | 8 | 9 |  | 41.99 |
| **9** | 1 | 2 | 3 | 4 | 5 | 6 | 7 | 8 | 9 | 40.88 |

**Table S11. Mean degree of niche overlapping for the four diatoms based on a threshold of abundance T=0.05.** The first column displays the number of dimensions considered simultaneously, columns 2 to 10 display the combinations of dimensions (i.e. 1=bathymetry, 2=nitrate, 3=phosphate, 4=silicate, 5=MLD, 6=temperature, 7=PAR, 8=salinity and 9=euphotic depth). The last column displays index D associated with the combination of environmental dimensions. D=0% when species niches are different and D=100% when species niches are identical; the higher the number of dimensions, the lower the value of index D. Only the combinations of environmental variables that minimise values of index D are displayed. The ecological niche of each species is displayed in Figure 3.

| **Number of dimensions** | **Combinations** | | | | | | | | | **Index D (%)** |
| --- | --- | --- | --- | --- | --- | --- | --- | --- | --- | --- |
| **1** | 9 |  |  |  |  |  |  |  |  | 59.61 |
| **2** | 6 | 9 |  |  |  |  |  |  |  | 42.65 |
| **3** | 6 | 8 | 9 |  |  |  |  |  |  | 33.05 |
| **4** | 6 | 7 | 8 | 9 |  |  |  |  |  | 26.02 |
| **5** | 5 | 6 | 7 | 8 | 9 |  |  |  |  | 20.85 |
| **6** | 3 | 5 | 6 | 7 | 8 | 9 |  |  |  | 17.36 |
| **7** | 1 | 3 | 5 | 6 | 7 | 8 | 9 |  |  | 15.09 |
| **8** | 1 | 2 | 3 | 5 | 6 | 7 | 8 | 9 |  | 13.28 |
| **9** | 1 | 2 | 3 | 4 | 5 | 6 | 7 | 8 | 9 | 12.02 |

**Table S12. Mean degree of niche overlapping for the four diatoms based on a threshold of abundance T=0.1.** The first column displays the number of dimensions considered simultaneously, columns 2 to 10 display the combinations of dimensions (i.e. 1=bathymetry, 2=nitrate, 3=phosphate, 4=silicate, 5=MLD, 6=temperature, 7=PAR, 8=salinity and 9=euphotic depth). The last column displays index D associated with the combination of environmental dimensions. D=0% when species niches are different and D=100% when species niches are identical; the higher the number of dimensions, the lower the value of index D. Only the combinations of environmental variables that minimise values of index D are displayed. The ecological niche of each species is displayed in Figure 3.

| **Number of dimensions** | **Combinations** | | | | | | | | | **Index D (%)** |
| --- | --- | --- | --- | --- | --- | --- | --- | --- | --- | --- |
| **1** | 8 |  |  |  |  |  |  |  |  | 55.19 |
| **2** | 1 | 8 |  |  |  |  |  |  |  | 32.07 |
| **3** | 1 | 8 | 9 |  |  |  |  |  |  | 21.63 |
| **4** | 1 | 5 | 6 | 8 |  |  |  |  |  | 14.64 |
| **5** | 1 | 5 | 6 | 8 | 9 |  |  |  |  | 10.05 |
| **6** | 1 | 5 | 6 | 7 | 8 | 9 |  |  |  | 7.21 |
| **7** | 1 | 2 | 5 | 6 | 7 | 8 | 9 |  |  | 5.89 |
| **8** | 1 | 2 | 4 | 5 | 6 | 7 | 8 | 9 |  | 5.17 |
| **9** | 1 | 2 | 3 | 4 | 5 | 6 | 7 | 8 | 9 | 4.67 |

**Table S13. Mean degree of niche overlapping for the four diatoms based on a threshold of abundance T=0.5.** The first column displays the number of dimensions considered simultaneously, columns 2 to 10 display the combinations of dimensions (i.e. 1=bathymetry, 2=nitrate, 3=phosphate, 4=silicate, 5=MLD, 6=temperature, 7=PAR, 8=salinity and 9=euphotic depth). The last column displays index D associated with the combination of environmental dimensions. D=0% when species niches are different and D=100% when species niches are identical; the higher the number of dimensions, the lower the value of index D. Only the combinations of environmental variables that minimise values of index D are displayed. The ecological niche of each species is displayed in Figure 3.

| **Number of dimensions** | **Combinations** | | | | | | | | | **Index D (%)** |
| --- | --- | --- | --- | --- | --- | --- | --- | --- | --- | --- |
| **1** | 8 |  |  |  |  |  |  |  |  | 13.61 |
| **2** | 1 | 8 |  |  |  |  |  |  |  | 0.92 |
| **3** | 1 | 8 | 9 |  |  |  |  |  |  | 0.25 |
| **4** | 1 | 6 | 8 | 9 |  |  |  |  |  | 0.07 |
| **5** | 1 | 6 | 7 | 8 | 9 |  |  |  |  | 0.02 |
| **6** | 1 | 4 | 6 | 7 | 8 | 9 |  |  |  | 0.01 |
| **7** | 1 | 3 | 4 | 6 | 7 | 8 | 9 |  |  | 0.00 |
| **8** | 1 | 2 | 3 | 4 | 6 | 7 | 8 | 9 |  | 0.00 |
| **9** | 1 | 2 | 3 | 4 | 5 | 6 | 7 | 8 | 9 | 0.00 |

**Table S14. Mean degree of niche overlapping for the four copepods based on a threshold of abundance T=0.** The first column displays the number of dimensions considered simultaneously, columns 2 to 8 display the combinations of dimensions (i.e. 1=bathymetry, 2=MLD, 3=temperature, 4=PAR, 5=salinity, 6=chlorophyll-a concentration and 7=euphotic depth) and the last column displays index D associated with the combination of environmental dimensions. D=0% when species niches are different and D=100% when species niches are identical; the higher the number of dimensions, the lower the value of index D. Only the combinations of environmental variables that minimise values of index D are displayed. The ecological niche of each copepod is shown in Figure 4.

| **Number of dimensions** | **Combinations** | | | | | | | **Index D (%)** |
| --- | --- | --- | --- | --- | --- | --- | --- | --- |
| **1** | 5 |  |  |  |  |  |  | 93.62 |
| **2** | 2 | 5 |  |  |  |  |  | 89.65 |
| **3** | 2 | 3 | 5 |  |  |  |  | 87.85 |
| **4** | 1 | 2 | 3 | 5 |  |  |  | 86.98 |
| **5** | 1 | 2 | 3 | 4 | 5 |  |  | 86.13 |
| **6** | 1 | 2 | 3 | 4 | 5 | 6 |  | 86.13 |
| **7** | 1 | 2 | 3 | 4 | 5 | 6 | 7 | 86.13 |

**Table S15. Mean degree of niche overlapping for the four copepods based on a threshold of abundance T=0.05.** The first column displays the number of dimensions considered simultaneously, columns 2 to 8 display the combinations of dimensions (i.e. 1=bathymetry, 2=MLD, 3=temperature, 4=PAR, 5=salinity, 6=chlorophyll-a concentration and 7=euphotic depth) and the last column displays index D associated with the combination of environmental dimensions. D=0% when species niches are different and D=100% when species niches are identical; the higher the number of dimensions, the lower the value of index D. Only the combinations of environmental variables that minimise values of index D are displayed. The ecological niche of each copepod is shown in Figure 4.

| **Number of dimensions** | **Combinations** | | | | | | | **Index D (%)** |
| --- | --- | --- | --- | --- | --- | --- | --- | --- |
| **1** | 2 |  |  |  |  |  |  | 41.55 |
| **2** | 2 | 3 |  |  |  |  |  | 30.70 |
| **3** | 1 | 2 | 3 |  |  |  |  | 23.04 |
| **4** | 1 | 2 | 3 | 5 |  |  |  | 18.96 |
| **5** | 1 | 2 | 3 | 5 | 7 |  |  | 15.77 |
| **6** | 1 | 2 | 3 | 4 | 5 | 7 |  | 13.66 |
| **7** | 1 | 2 | 3 | 4 | 5 | 6 | 7 | 13.30 |

**Table S16. Mean degree of niche overlapping for the four copepods based on a threshold of abundance T=0.1.** The first column displays the number of dimensions considered simultaneously, columns 2 to 8 display the combinations of dimensions (i.e. 1=bathymetry, 2=MLD, 3=temperature, 4=PAR, 5=salinity, 6=chlorophyll-a concentration and 7=euphotic depth) and the last column displays index D associated with the combination of environmental dimensions. D=0% when species niches are different and D=100% when species niches are identical; the higher the number of dimensions, the lower the value of index D. Only the combinations of environmental variables that minimise values of index D are displayed. The ecological niche of each copepod is shown in Figure 4.

| **Number of dimensions** | **Combinations** | | | | | | | **Index D (%)** |
| --- | --- | --- | --- | --- | --- | --- | --- | --- |
| **1** | 2 |  |  |  |  |  |  | 36.96 |
| **2** | 2 | 5 |  |  |  |  |  | 17.37 |
| **3** | 1 | 2 | 5 |  |  |  |  | 10.78 |
| **4** | 1 | 2 | 3 | 5 |  |  |  | 6.75 |
| **5** | 1 | 2 | 3 | 5 | 7 |  |  | 5.44 |
| **6** | 1 | 2 | 3 | 4 | 5 | 7 |  | 4.87 |
| **7** | 1 | 2 | 3 | 4 | 5 | 6 | 7 | 4.77 |

**Table S17. Mean degree of niche overlapping for the four copepods based on a threshold of abundance T=0.5.** The first column displays the number of dimensions considered simultaneously, columns 2 to 8 display the combinations of dimensions (i.e. 1=bathymetry, 2=MLD, 3=temperature, 4=PAR, 5=salinity, 6=chlorophyll-a concentration and 7=euphotic depth) and the last column displays index D associated with the combination of environmental dimensions. D=0% when species niches are different and D=100% when species niches are identical; the higher the number of dimensions, the lower the value of index D. Only the combinations of environmental variables that minimise values of index D are displayed. The ecological niche of each copepod is shown in Figure 4.

| **Number of dimensions** | **Combinations** | | | | | | | **Index D (%)** |
| --- | --- | --- | --- | --- | --- | --- | --- | --- |
| **1** | 5 |  |  |  |  |  |  | 16.40 |
| **2** | 1 | 5 |  |  |  |  |  | 1.16 |
| **3** | 1 | 3 | 5 |  |  |  |  | 0.79 |
| **4** | 1 | 3 | 5 | 7 |  |  |  | 0.53 |
| **5** | 1 | 3 | 4 | 5 | 7 |  |  | 0.40 |
| **6** | 1 | 2 | 3 | 4 | 5 | 7 |  | 0.33 |
| **7** | 1 | 2 | 3 | 4 | 5 | 6 | 7 | 0.31 |

**Supplementary Figures.**

**Figure S1. Species chromatograms of four diatom species without the application of the second-order simple moving average and the standardisation of the abundance between 0 and 1.** Species chromatograms for (**a**) *Paralia sulcata*, (**b**) *Skeletonema costatum*, (**c**) *Rhizosolenia styliformis* and (**d**) *Rhizosolenia bergonii*. In **a**-**d**, each column represents the mean species abundance along nine environmental dimensions (i.e. bathymetry, nitrate, phosphate and silicate concentration, MLD, temperature, PAR, salinity and euphotic depth). Species abundance in each category (colour in the cells) was assessed by estimating the abundance of the 5% of the highest values available in a category if at least 1 CPR sample was available in that category. The Y axis corresponds to the 50 categories standardised between 0 and 1. This axis represents all values taken by an environmental variable between 0 and 1 from the lowest (bottom category) to the highest (top category). Colours represent the mean species abundance (without standardisation between 0 and 1) in each category. High abundance values are in red and low values in blue.

**Figure S2. Species chromatograms of four copepods without the application of the second-order simple moving average and the standardisation of the abundance between 0 and 1.** Species chromatograms for (**a**) *Temora longicornis*, (**b**) *Clausocalanus* spp*.*, (**c**) *Calanus finmarchicus* and (**d**) *Calanus helgolandicus*. In **a**-**d**, each column represents the mean species abundance along seven environmental dimensions (i.e. bathymetry, MLD, temperature, PAR, salinity, chlorophyll-a concentration and euphotic depth). Species abundance in each category (colour in the cells) was assessed by estimating the abundance of the 5% of the highest values available in a category if at least 1 CPR sample was available in that category. The Y axis corresponds to the 50 categories standardised between 0 and 1. This axis represents all values taken by an environmental variable between 0 and 1 from the lowest (bottom category) to the highest (top category). Colours represent the mean species abundance (without standardisation between 0 and 1) in each category. High abundance values are in red and low values in blue.

**Figure S3. Species chromatograms of four diatom species with the application of the second-order simple moving average but without the standardisation of the abundance between 0 and 1.** Species chromatograms for (**a**) *Paralia sulcata*, (**b**) *Skeletonema costatum*, (**c**) *Rhizosolenia styliformis* and (**d**) *Rhizosolenia bergonii*. In **a**-**d**, each column represents the mean species abundance along nine environmental dimensions (i.e. bathymetry, nitrate, phosphate and silicate concentration, MLD, temperature, PAR, salinity and euphotic depth). Species abundance in each category (colour in the cells) was assessed by estimating the abundance of the 5% of the highest values available in a category if at least 1 CPR sample was available in that category. The Y axis corresponds to the 50 categories standardised between 0 and 1. This axis represents all values taken by an environmental variable between 0 and 1 from the lowest (bottom category) to the highest (top category). Colours represent the mean species abundance (without standardisation between 0 and 1) in each category. High abundance values are in red and low values in blue.

**Figure S4. Species chromatograms of four copepods with the application of the second-order simple moving average but without the standardisation of the abundance between 0 and 1.** Species chromatograms for (**a**) *Temora longicornis*, (**b**) *Clausocalanus* spp*.*, (**c**) *Calanus finmarchicus* and (**d**) *Calanus helgolandicus*. In **a**-**d**, each column represents the mean species abundance along seven environmental dimensions (i.e. bathymetry, MLD, temperature, PAR, salinity, chlorophyll-a concentration and euphotic depth). Species abundance in each category (colour in the cells) was assessed by estimating the abundance of the 5% of the highest values available in a category if at least 1 CPR sample was available in that category. The Y axis corresponds to the 50 categories standardised between 0 and 1. This axis represents all values taken by an environmental variable between 0 and 1 from the lowest (bottom category) to the highest (top category). Colours represent the mean species abundance (without standardisation between 0 and 1) in each category. High abundance values are in red and low values in blue.

**Figure S5. Species chromatograms of four diatom species with the application of the second-order simple moving average and the standardisation of the abundance between 0 and 1.** Species chromatograms for (**a**) *Paralia sulcata*, (**b**) *Skeletonema costatum*, (**c**) *Rhizosolenia styliformis* and (**d**) *Rhizosolenia bergonii*. In **a**-**d**, each column represents the species abundance along nine environmental dimensions (i.e. bathymetry, nitrate, phosphate and silicate concentration, MLD, temperature, PAR, salinity and euphotic depth). Species abundance in each category (colour in the cells) was assessed by estimating the abundance of the 5% of the highest values available in a category if at least 1 CPR sample was available in that category. The Y axis corresponds to the 50 categories standardised between 0 and 1. This axis represents all values taken by an environmental variable between 0 and 1 from the lowest (bottom category) to the highest (top category). Colours represent the mean species abundance (standardised between 0 and 1) in each category. High abundance values are in red and low values in blue.

**Figure S6.** **Species chromatograms of four copepods with the application of the second-order simple moving average and the standardisation of the abundance between 0 and 1.** Species chromatograms for (**a**) *Temora longicornis*, (**b**) *Clausocalanus* spp*.*, (**c**) *Calanus finmarchicus* and (**d**) *Calanus helgolandicus*. In **a**-**d**, each column represents the mean species abundance along seven environmental dimensions (i.e. bathymetry, MLD, temperature, PAR, salinity, chlorophyll-a concentration and euphotic depth). Species abundance in each category (colour in the cells) was assessed by estimating the abundance of the 5% of the highest values available in a category if at least 1 CPR sample was available in that category. The Y axis corresponds to the 50 categories standardised between 0 and 1. This axis represents all values taken by an environmental variable between 0 and 1 from the lowest (bottom category) to the highest (top category). Colours represent the mean species abundance (standardised between 0 and 1) in each category. High abundance values are in red and low values in blue.

**Figure S7. Total number of samples (log_10_ transformed) available in each category.**

**Supplementary Figure S8. Species chromatograms of the 14 pseudo-species.** Each panel displays the three-dimensional ecological niche of a pseudo-species (i.e. virtual species). In **a**-**n**, each column represents the mean pseudo-species abundance along three environmental dimensions (i.e. p_1_, p_2_ and p_3_). Pseudo-species abundance in each category (colour in the cells) was assessed by estimating the abundance of the 5% of the highest values available in a category if at least 1 sample was available in that category. The Y axis corresponds to the 50 categories standardised between 0 and 1. This axis represents all values taken by an environmental variable between 0 and 1 from the lowest (bottom category) to the highest (top category). Colours represent the mean species abundance (standardised between 0 and 1) in each category. High abundance values are in red and low values in blue.

**Supplementary Figure S9. Three-dimensional ecological niches of the 14 pseudo-species display with the *hypervolume* package.** Each panel displays the three-dimensional ecological niche of a pseudo-species as a set of pair plots of the pseudo-species abundance repartition along the three environmental dimensions p_1_, p_2_ and p_3_. Niche hypervolumes were delineated by means of a Gaussian kernel density estimation.

**
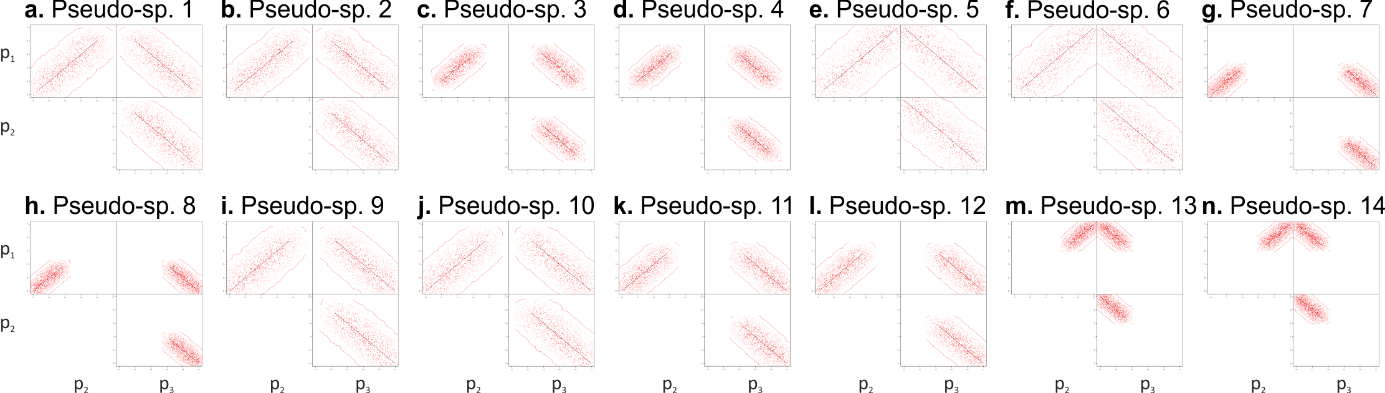
**

**Supplementary Figure S10. Three-dimensional ecological niches of the 14 pseudo-species display with the *hypervolume* package.** Each panel displays the three-dimensional ecological niche of a pseudo-species as a set of pair plots of the pseudo-species abundance repartition along the three environmental dimensions p_1_, p_2_ and p_3_. Niche hypervolumes were delineated by means of a one-class support vector machine.

**
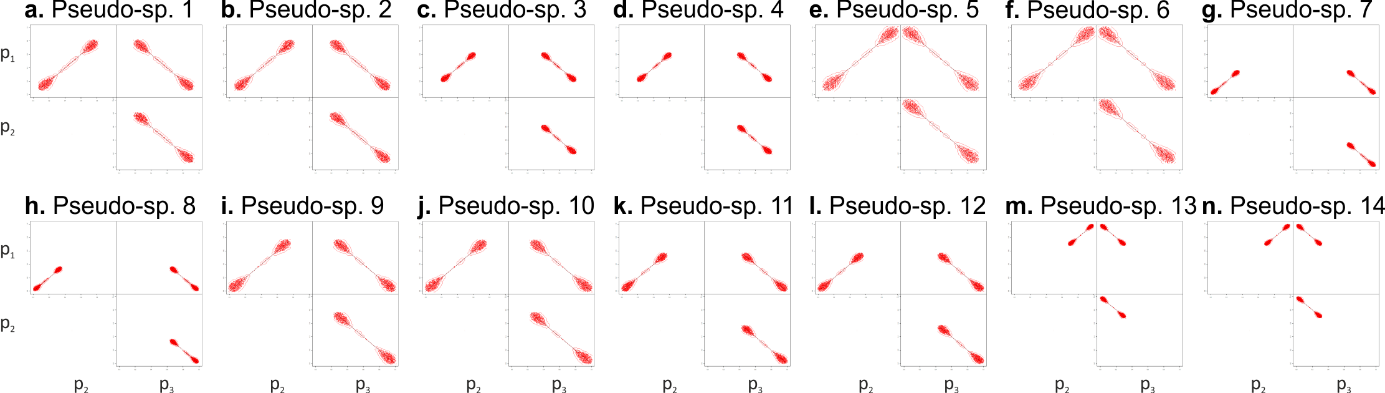
**

**References**

Barnard, B., Batten, S. D., Beaugrand, G., Buckland, C., Conway, D. V. P., Edwards, M., Finlayson, J., Gregory, L. W., Halliday, N. C., John, A. W. G., Johns, D. G., Johnson, A. D., Jonas, T. D., Lindley, J. A., Nyman, J., Pritchard, P., Reid, P. C., Richardson, A. J., Saxby, R. E., … Wright, J. C. (2004). Continuous Plankton Records: Plankton Atlas of the North Atlantic Ocean (1958-1999). II. Biogeographical charts. *Marine Ecology Progress Series*, *Supplement*, 11–75.

Batten, S. D., Clark, R., Flinkman, J., Hays, G., John, E., John, A. W. G., Jonas, T., Lindley, J. A., Stevens, D. P., & Walne, A. (2003). CPR sampling: The technical background, materials and methods, consistency and comparability. *Progress in Oceanography*, *58*(2–4), 193–215. https://doi.org/10.1016/j.pocean.2003.08.004

Hays, G. C., & Warner, A. J. (1993). Consistency of Towing Speed and Sampling Depth for the Continuous Plankton Recorder. *Journal of the Marine Biological Association of the United Kingdom*, *73*(4), 967–970. https://doi.org/10.1017/S0025315400034846

Helaouët, P. (2021). *Marine Biological Association of the UK (MBA) (2021): Continuous Plankton Recorder data. The Archive for Marine Species and Habitats Data (DASSH).* https://doi.org/10.17031/1708

Hutchinson, G. E. (1957). Concluding remarks. *Cold Spring Harbor Symposia on Quantitative Biology*, *22*, 415–427.

Hutchinson, G. E. (1978). *An introduction to population ecology* (Yale University Press).

Kléparski, L., Beaugrand, G., & Edwards, M. (2021). Plankton biogeography in the North Atlantic Ocean and its adjacent seas: Species assemblages and environmental signatures. *Ecology and Evolution*, *11*(10), 5135–5149. https://doi.org/10.1002/ece3.7406

Morel, A., Huot, Y., Gentili, B., Werdell, P. J., Hooker, S. B., & Franz, B. A. (2007). Examining the consistency of products derived from various ocean color sensors in open ocean (Case 1) waters in the perspective of a multi-sensor approach. *Remote Sensing of Environment*, *111*(1), 69–88. https://doi.org/10.1016/j.rse.2007.03.012

Swinehart, D. F. (1962). The Beer-Lambert law. *Journal of Chemical Education*, *39*(7), 333–335.

Wackernagel, H. (1995). *Multivariate geostatistics. An introduction with applications.* Springer-Verlag.

Warner, A. J., & Hays, G. C. (1994). Sampling by the continuous plankton recorder survey. *Progress in Oceanography*, *34*(2–3), 237–256. https://doi.org/10.1016/0079-6611(94)90011-6
